# Abca4, mutated in Stargardt disease, is required for structural integrity of cone outer segments

**DOI:** 10.1242/dmm.052052

**Published:** 2025-01-10

**Authors:** John J. Willoughby, Abbie M. Jensen

**Affiliations:** Biology Department, University of Massachusetts, Amherst, MA 01003, USA

**Keywords:** Photoreceptor, Macular degeneration, Phagocytosis, Zebrafish

## Abstract

Stargardt disease (STGD), the leading cause of inherited childhood blindness, is primarily caused by mutations in the *ABCA4* gene; yet, the underlying mechanisms of photoreceptor degeneration remain elusive, partly due to limitations in existing animal disease models. To expand our understanding, we mutated the human *ABCA4* paralogues *abca4a* and *abca4b* in zebrafish, which has a cone-rich retina. Our study unveiled striking dysmorphology and elongation of cone outer segments (COS) in *abca4a;abca4b* double mutants, alongside reduced phagocytosis by the retinal pigmented epithelium (RPE). We report that zebrafish Abca4 protein forms a distinctive stripe along the length of COS, suggesting a potential structural role. We further show that, in wild-type zebrafish, outer segments of cone cells constitutively present externalized phosphatidylserine, an apoptotic ‘eat-me’ signal, and that this pattern is disrupted in *abca4a*;*abca4b* double mutants, potentially contributing to reduced RPE phagocytic activity. More broadly, constitutive presentation of the ‘eat-me’ signal by COS − if conserved in humans – might have important implications for other retinal degenerative diseases, including age-related macular degeneration. Our zebrafish model provides novel insights into cone dysfunction and presents a promising platform for unraveling the mechanisms of STGD pathogenesis and advancing therapeutic interventions.

## Teaser

**Summary:** A zebrafish model of Stargardt disease, a juvenile-onset macular degeneration, provides insight into the disease mechanisms.

## INTRODUCTION

Stargardt disease (STGD), also known as juvenile-onset macular degeneration, is the most common cause of macular degeneration in children, with central vision loss often beginning in the first decade of life. STGD affects central vision largely due to loss of cone photoreceptor function in that region. High acuity visual activities, such as reading and recognizing faces, are severely disrupted, although peripheral vision can be largely preserved. No treatments are currently available to delay or cure STGD. Mutations in the *ABCA4* gene are most commonly associated with STGD and with progressive cone dystrophies (Allikmets et al., 1997; reviewed by Gill et al., 2019; Cremers et al., 2020). Recessive *ABCA4* mutations are less often associated with retinitis pigmentosa, cone-rod dystrophy and macular dystrophy with flecks (Rozet et al., 1999; Klevering et al., 2002; Valverde et al., 2007; Zahid et al., 2013).

The Abca4 protein has first been described as localizing uniquely to the rims of the outer segment membrane disks in mouse rods (Sun and Nathans, 1997) and proposed to play a vital role in transporting the all-*trans* retinal (ATR) from photoreceptor disk membranes towards the neighboring retinal pigmented epithelium (RPE) for the eventual regeneration of bleached visual pigments (Beharry et al., 2004). It has been postulated that, in the absence of ABCA4 function, N-retinylidene-phosphatidylethanolamine (NRPE) – a reversible covalent adduct of ATR and phosphatidylethanolamine (PE) – accumulates in photoreceptor outer segment membranes, so that phagocytosis by RPE of the NRPE-laden outer segment material becomes increasingly toxic to the RPE and, consequently, photoreceptors degenerate from lost RPE support (Radu et al., 2004; see review by Tsybovsky et al., 2010). More recently, Abca4 has been shown to prevent the accumulation of toxic excess 11-*cis*-retinal in photoreceptors (Quazi and Molday, 2014); thus, loss of ABCA4 function could additionally and directly impact photoreceptor viability. RPE lipofuscin, composed predominantly of retinoid byproducts, is observed in patients diagnosed with STGD and also accumulates in *abca4* deficit mice, in which only modest rod degeneration is observed (Weng et al., 1999; Mata et al., 2000; Lenis et al., 2018). The phenotypic difference between humans carrying mutations in *ABCA4* and *abca4*^−/−^ mice might be because, in mice, ∼97% of photoreceptors are rods (Jeon et al., 1998), while the human macula is comparatively cone-rich and the fovea comprises ∼100% cones (Curcio et al., 1990).

An animal model that presents features of STGD macular degeneration, including cone photoreceptor and/or RPE dysfunction, is crucial for pursuing translational research. Therefore, to create a more informative disease model of cone dysfunction needed to understand the molecular and cellular mechanisms that contribute to STGD, we introduced mutations to the *abca4* genes (*abca4a*, *abca4b*) in zebrafish, an animal with a cone-rich retina that is similar to the human macula (Fadool, 2003), and examined photoreceptors and RPE in single and double *abca4* zebrafish mutants.

## RESULTS

### Creation of the *abca4* zebrafish mutants

**Fig. 1.**
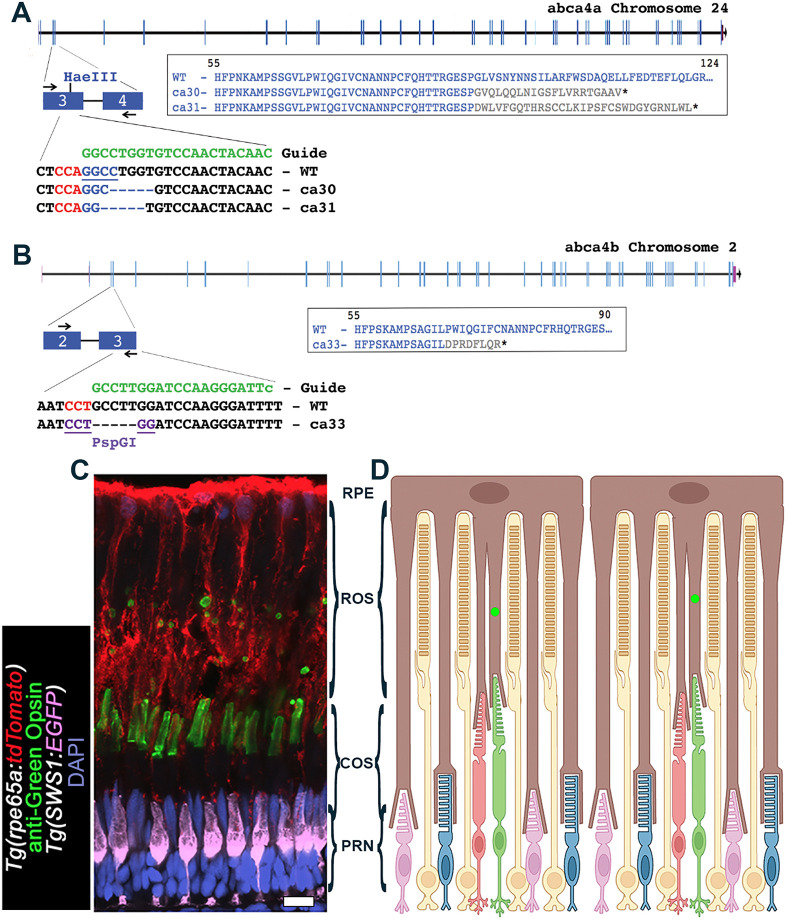
**Generation of zebrafish *abca4* mutants by using CRISPR/Cas9.** (A) Shown is a sequence in exon 3 of the *abca4a* gene on chromosome 24 (NCBI: Gene ID: 798993) that was targeted with a synthetic guide RNA. Two mutant alleles – *abca4a^ca30^* (ca30) and abca4a^ca31^ (ca31) − that disrupt a HaeIII restriction site, were identified and mutant lines established. Both mutant alleles disrupt the reading frame through addition of 21 (*ca30*) or 31 (*ca31*) missense residues, thereby truncating at a position equivalent to amino acid (aa) 112 or 122 in WT. (B) Shown is a sequence in exon 3 of the *abca4b* gene on chromosome 2 (NCBI: Gene ID: 555506) that was targeted with a synthetic guide RNA. The single mutant allele *abca4b^ca33^* (ca33) that creates a novel PspGI restriction site, was identified and the mutant line established. Allele *ca33* is predicted to disrupt the reading frame through addition of eight missense aa, before truncating at a position equivalent to aa 75 in WT. Synthetic guide RNA sequences are shown in green; Cas9 protospacer adjacent motif (PAM) sequences are shown in red. (C) Confocal *z*-projection (*z*=2.82 µm) of the photoreceptor layer in the retina of *Tg(rpe65a:tdTomato)*;*Tg(SWS1:EGFP)* zebrafish aged 1 year. The retinal pigmented epithelium (RPE) expressing tdTomato (red), UV cones express EGFP (pseudo-colored violet). Green cone outer segment (COS) regions were stained for Green Opsin (green); phagosomes of green COS ingested by RPE microvilli are visible as green ‘spheres’ distal to green COS, which are transported to the RPE cell bodies for digestion. Nuclei are shown in blue (DAPI). Scale bar: 10 µm. (D) Schematic of the photoreceptor layer showing photoreceptor types (yellow rods, green cones, red cones, blue cones, UV cones), RPE (brown), rod outer segment (ROS) region, cone outer segment (COS) region and photoreceptor nuclei (PRN), i.e. outer nuclear region (ONL) region in zebrafish retina. Green dots indicate phagosomes of green COS ingested by RPE microvilli.

To create a zebrafish model of STGD, we targeted the two zebrafish paralogues *abca4a* and *abca4b* of human *ABCA4*. We used CRISPR/Cas9 and designed gRNAs to mutate a sequence in exon 3 in *abca4a* and *abca4b* (Fig. 1). We recovered two *abca4a* mutant alleles, i.e. *abca4a^ca30^* and *abca4a^ca31^*, that disrupt the reading frame, and both alleles comprise a destroyed HaeIII restriction site present in the wild-type (WT) allele (Fig. 1A). We also recovered mutant allele *abca4b^ca33^* comprising a disrupted reading frame, which generated a PspG1 restriction site that is absent in the WT allele (Fig. 1B). Sequence analysis of mRNA by RT-PCR verified the mutations seen in gDNA of *abca4a* and *abca4b* mutant alleles (data not shown). All mutations were expected to produce null alleles (also confirmed by mRNA sequence analysis), resulting in severely truncated proteins, when resulting transcripts were translated (Fig. 1). The *abca4* mutant alleles were then crossed into the background of two transgenic fluorescent reporter lines − *Tg(rpe65a:tdTom)* for retinal pigmented epithelium (RPE) and *Tg(SWSW1:EGFP)* for short-wave (UV) cone photoreceptors (Fig. 1C) (see Materials and Methods; Takechi et al., 2003). Views of the photoreceptor layer are provided in Fig. 1C,D, with the RPE at the top and the outer nuclear layer (photoreceptor nuclei) at the bottom. This view and orientation of the photoreceptor layer are similar in all subsequent immunohistochemistry images. All genotypes are in the *albino* (*alb^−/−^*) background (Dooley et al., 2013), as dense RPE melanin obscures the fluorescent visualization of photoreceptor outer segments.

### Localization of Abca4 protein in the zebrafish retina

**Fig. 2.**
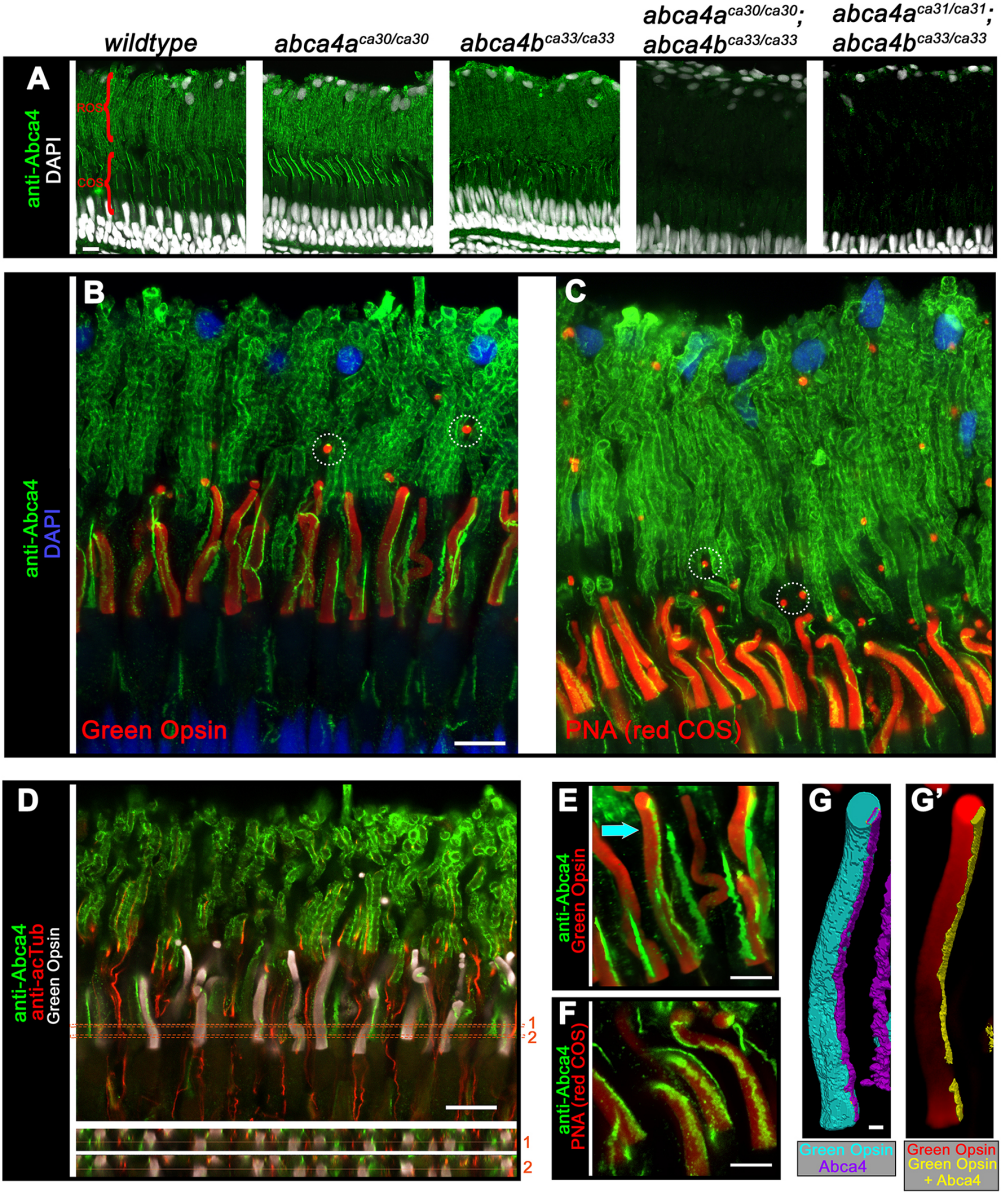
**Abca4 protein location.** (A) Confocal *z*-projections (*z*=2.5 µm) of the photoreceptor layer stained for Abca4 (green) in retinas obtained from 2-month-old wild-type, or *abca4a^ca30/ca30^*, *abca4b^ca33/ca33^*, *abca4a^ca30/ca30^;abca4b^ca33/ca33^* and *abca4a^ca31/ca31^;abca4b^ca33/ca33^* mutant zebrafish. Nuclei (white) were labeled with DAPI. Regions of rod outer segments (ROS) and cone outer segment (COS) are indicated by red brackets. (B,C) Confocal *z*-projections (*z*=5.4 µm) of photoreceptor layers from 4-month-old wild-type zebrafish co-stained for Abca4 (green) and either Green Opsin (red) or PNA (red) (B or C, respectively). Nuclei (blue) were labeled with DAPI. Encircled areas show examples of COS phagosomes stained for Abca4. (D) Confocal *xy*-slice view of the photoreceptor layer obtained from a 2-month-old wild-type fish, co-stained for Abca4 (green), acetylated tubulin (red) and Green Opsin (white). Numbers in red (on right) indicate the two *xz*-slice views (z=5.3 µm) that are shown below. (E,F) 3D-volume projections of COS obtained from 4-month-old wild-type zebrafish co-stained for Abca4 (green) and either green opsin (red; E) or PNA (red; F). Arrow in E indicates the COS that is volume-filled rendered in G and G′. (G,G′) To reveal protein colocalization, the single green COS, indicated by the arrow in E, is shown as a volume-filled rendering of Abca4 and Green Opsin antibody staining. Staining for Green Opsin (cyan) and Abca4 (purple) is shown in G; G′ shows staining for Green Opsin (red) as well as colocalized Green Opsin and Abca4 (yellow), with the latter largely being buried under area shown in purple in G. Scale bars: 10 µm (A,B,D), 5 µm (E,F), 1 µm (G).

Because cellular and subcellular protein localization provides insight into protein function, we raised a custom rabbit polyclonal Abca4 antibody against a conserved peptide within Abca4a and Abca4b at amino acid positions 152-165. In *wt* retina, this anti-Abca4 antibody labeled rod outer segments (ROS) as well as cone outer segments (COS) (see Fig. 2A). Immunostaining of *abca4a* mutant retina resulted in Abca4 levels similar to those in WT, while immunostaining of *abca4b* mutant or double mutant retinas (i.e. *abca4a^ca30/ca30^*;*abca4^ca33/ca33^* or *abca4a^ca31/ca31^*;*abca4^ca33/ca33^*) showed overall reduced or absent Abca4 levels, respectively (Fig. 2A).

To confirm that Abca4 localized to COS, we double-labeled *wt* retina with anti-Abca4 antibodies (green) and cone markers, green COS labeled with anti-Green Opsin antibodies (red, Fig. 2B), and red COS labeled with peanut agglutinin (PNA) (red, Fig. 2C). The Abca4 protein extended as a stripe along the length of COS, and presented as a ladder-like structure in ROS. Single puncta were associated with phagosomes shed by COS (Fig. 2B,C; encircled areas). Photoreceptor outer segments are modified primary cilia; so, to examine whether the Abca4 stripe in COS colocalizes with the COS ciliary axoneme, we double-stained *wt* retina for Abca4 (green) and acetylated tubulin (red). Fig. 2D shows that these two structures did not colocalize. High-magnification and higher-resolution 3D-volume projections of anti-Abca4 and COS markers provided more morphological detail of Abca4 and its association with the COS membrane (Fig. 2E,F). To determine whether Abca4 is integral to the COS membrane, we examined the extent of Abca4 colocalization with the membrane protein medium-wave-sensitive opsin (Opn1mw; hereafter referred to as Green Opsin) that is integral to COS. We did this by using the single green COS (indicated by the arrow in Fig. 2E) and applying 3D volume-fill rendering of Green Opsin and Abca4 (Fig. 2G; visualized in cyan and purple, respectively). The portion of Abca4 that colocalized with Green Opsin is volume-fill rendered as yellow (see Fig. 2G′), and underneath the ‘Abca4-alone’ portion (purple; Fig. 2G) together with Green Opsin (red; Fig. 2G′). To gain a quantitative sense of the extent of Abca4 and Green opsin colocalization in COS, we analyzed a population of green COS, rendered as shown in Fig. 2G,Gʹ and calculated the percentage of Abca4-positive pixels that are also Green Opsin positive and found a mean colocalization of 53.5% with wide variation (standard deviation=27.9). Additional volume views of anti-Abca4 labeling of the COS region are provided in Fig. S1.

### Cone photoreceptor morphology is abnormal in the *abca4* mutant zebrafish

**Fig. 3.**
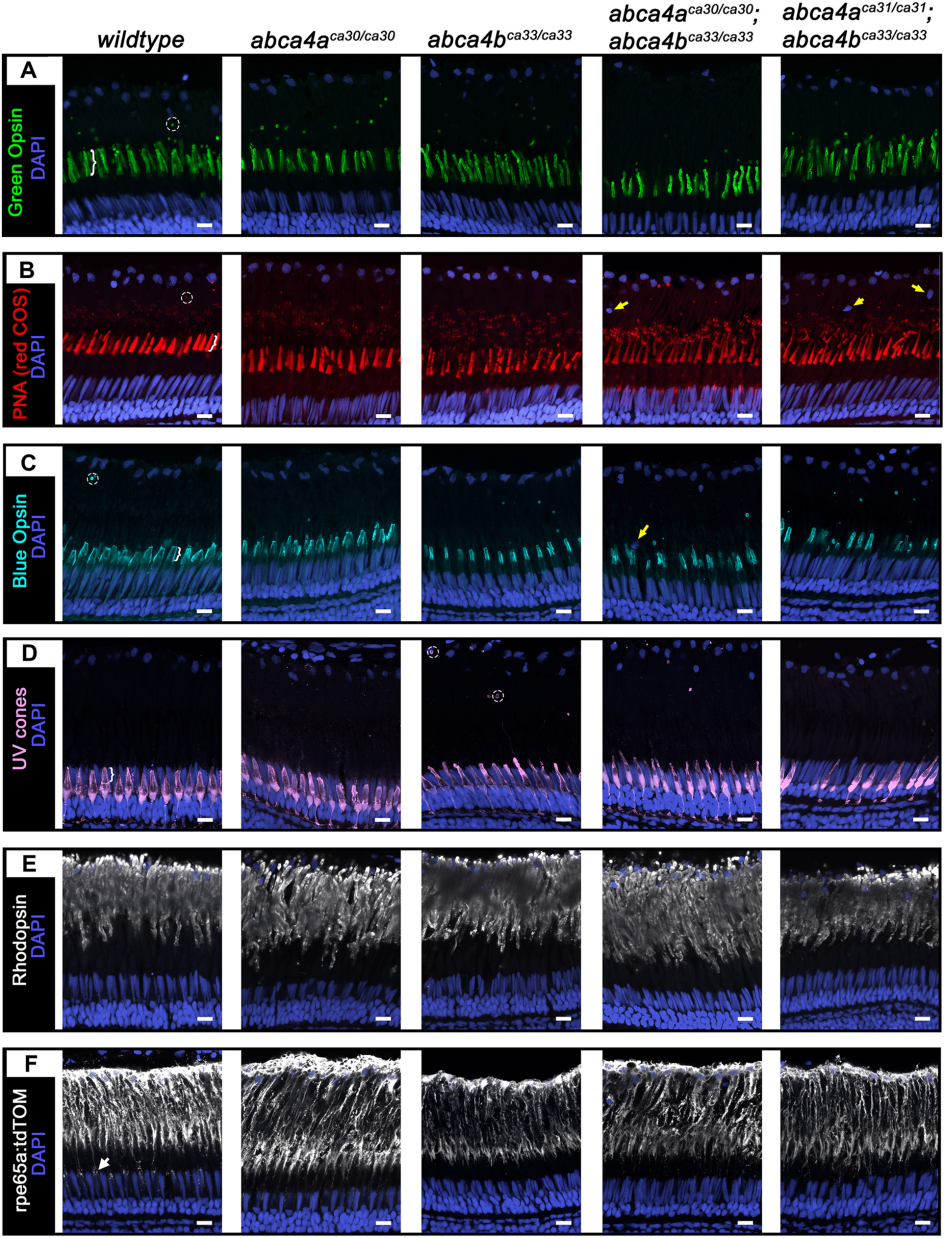
**Morphology of COS, ROS and RPE in retinas of 2-month-old zebrafish.** (A-F) Confocal *z*-projections of COS, ROS and RPE in retinas obtained from wild-type or *abca4a^ca30/ca30^*, *abca4b^ca33/ca33^*, *abca4a^ca30/ca30^*;*abca4b^ca33/ca33^* and *abca4a^ca31/ca31^*;*abca4b^ca33/ca33^* mutant zebrafish. (A) Green COS stained for Green Opsin (green; *z*=3 µm), (B) red COS stained for PNA (red; *z*=3 µm), (C) blue COS stained for Blue Opsin (cyan; *z*=3.4 µm), (D) UV cones expressing EGFP stained for GFP (violet; *z*=4.4 µm), (E) ROS stained for rhodopsin (white; *z*=1.9 µm) and (F) RPE expressing tdTomato stained for RFP (white, *z*=2.4 µm). Counterstaining for nuclei is shown in dark blue (DAPI). White arrow in F (wild-type panel) indicates distal RPE processes around UV COS (not visible). Encircled areas show examples of phagosomes in retinas of wild-type and *abca4b^ca33/^*^ca33^ fish. White brackets in wild-type panels A-D indicate COS. Yellow arrows indicate examples of non RPE (‘mystery’) nuclei in B and C. Scale bars: 10 µm.

The cone-rich retina of zebrafish provides an opportunity to view the effects of *abca4* mutations on cone photoreceptors and gain mechanistic insight into STGD. We examined the photoreceptor layer in retina obtained from juvenile (2-month-old) *wt* fish, and *abca4a^−/−^*, *abca4b^−/−^* and *abca4a^−/−^*;*abca4b^−/−^* double mutants (Fig. 3). COS were stained for either Green or Blue Opsin (hereafter referred to as green COS or blue COS, respectively) (Fig. 3A,C), or labeled with PNA (hereafter referred to as red COS), which also labels the base of ROS (Fig. 3B). For labeling of blue COS, we used antibody against short-wave-sensitive opsin (Opn1sw; hereafter referred to as Blue Opsin) (Fig. 3C). UV cones were visualized by expression of enhanced green fluorescent protein (EGFP) [*Tg(SWS1:EGFP)*, Fig. 3D]. White brackets in Fig. 3A-D indicate an example of each COS subtype. ROS were labeled by anti-rhodopsin antibodies (Fig. 3E). RPE was visualized by expression of tdTomato [*Tg(rpe65a:tdTomato)*, Fig. 3F]. The morphology of COS subtypes (green, red, blue and UV), ROS and RPE was similar between *wt* fish and *abca4a^ca30/ca30^* mutants. At this early age, COS abnormalities were already apparent in the retina of *abca4b^ca33/ca33^*, *abca4a^ca30/ca30^*;*abca4b^ca33/ca33^* and *abca4a^ca31/ca31^*;*abca4b^ca33/ca33^* double mutants compared to retina of *wt* fish. In *wt* retina, green COS were straight and tubular comprising smooth contours with slightly tapered tips, and numerous shed green phagosomes were visible (see encircled area in Fig. 3A), whereas those in *abca4b^ca33/ca33^* and double mutants appeared jagged and crumpled, with fewer shed phagosomes (Fig. 3A). In the *wt* retina, the shorter red COS with more-pointed tips were uniformly labeled by PNA and we observed several shed red phagosomes. However, in *abca4b^ca33/ca33^* and double mutants PNA labeling appeared discontinuous, sometimes visible as stripes, and many COS were noticeably longer (Fig. 3B). In the *wt* retina, blue COS were squat and flat-topped, and the entire plasma membrane was lightly labeled for Blue Opsin, whereas, in *abca4b^ca33/ca33^* and double mutants, blue COS were narrow and of a tubular shape with rounded tips (Fig. 3C). In the *wt* retina, UV COS were short and cone-shaped but in *abca4b^ca33/ca33^* and double mutants UV COS were longer and more tubular. Moreover, while we did not observe UV phagosomes in the *wt* retina, they were present in the *abca4* mutant retinas, with most of them located within or near to the cell bodies of the RPE (Fig. 3D). ROS were much longer than COS and positioned above COS, with their tips adjacent to the RPE apical surface; however, they appeared to be similar in all genotypes (Fig. 3E). The RPE sends processes deep into the outer segment region to support COS function, and the RPE appeared to be similar in all genotypes (Fig. 3F). Occasionally, we observed nuclei in the photoreceptor outer segment region that do not express the *rpe65a:tdTomato* transgene (yellow arrows, Fig. 3B,C).

**Fig. 4.**
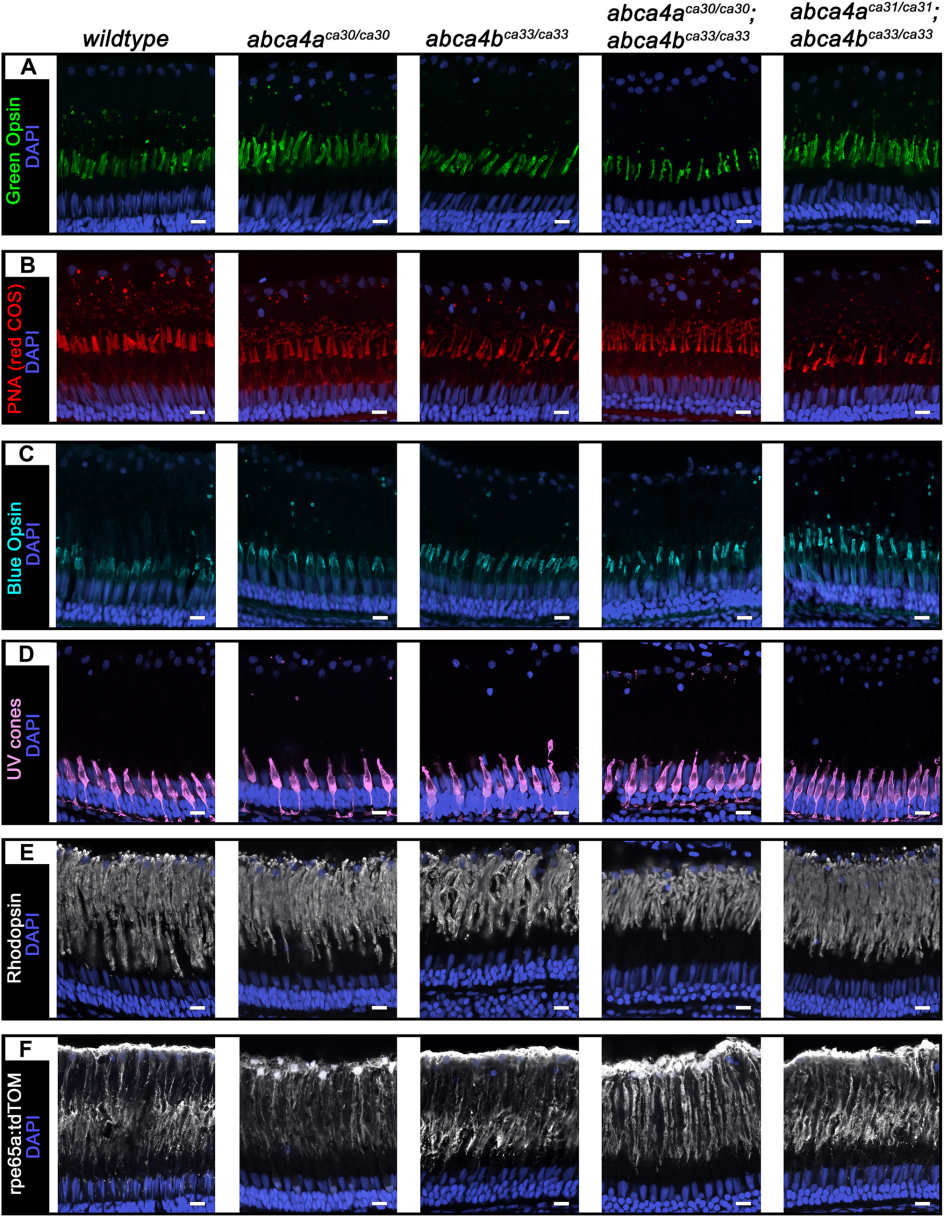
**Morphology of COS, ROS and RPE in retinas of 1-year-old zebrafish.** (A-F) Confocal *z*-projections of COS, ROS and RPE in retinas obtained from wild-type or *abca4a^ca30/ca30^*, *abca4b^ca33/ca33^*, *abca4a^ca30/ca30^*;*abca4b^ca33/ca33^* and *abca4a^ca31/ca31^*;*abca4b^ca33/ca33^* mutant zebrafish. (A) Green COS stained for Green Opsin (green, *z*=4.3 µm), (B) red COS stained for PNA (red, *z*=4.3 µm), (C) blue COS stained for Blue Opsin (cyan, *z*=4.3 µm), (D) UV cones expressing EGFP stained for GFP (violet, *z*=4.4 µm;), (E) ROS stained for -rhodopsin (white, *z*=1.9 µm) and (F) RPE expressing tdTomato stained for RFP (white, *z*=2.4 µm). Nuclei (blue) were labeled with DAPI. Scale bars: 10 µm.

Because STGD results in progressive loss of vision, we examined outer segments and RPE in the retinas of 1-year-old (middle aged) *wt*, *abca4a^ca30/ca30^*, *abca4b^ca33/ca33^* and *abca4a^−/−^*;*abca4b^−/−^* double mutant zebrafish (Fig. 4). The morphology of green COS (Fig. 4A), red COS (Fig. 4B), blue COS (Fig. 4C), UV COS (Fig. 4D), ROS (Fig. 4E) and RPE (Fig. 4F) in these middle aged fish was similar to that of 2-month-old fish, but differences regarding COS morphology and phagosome number are more apparent in retinas of *wt* and *abca4b^ca33/ca33^* or *abca4a^−/−^*;*abca4b^−/−^* double mutant fish.

### COS are longer and phagocytosis is disrupted in *abca4* mutant zebrafish

**Fig. 5.**
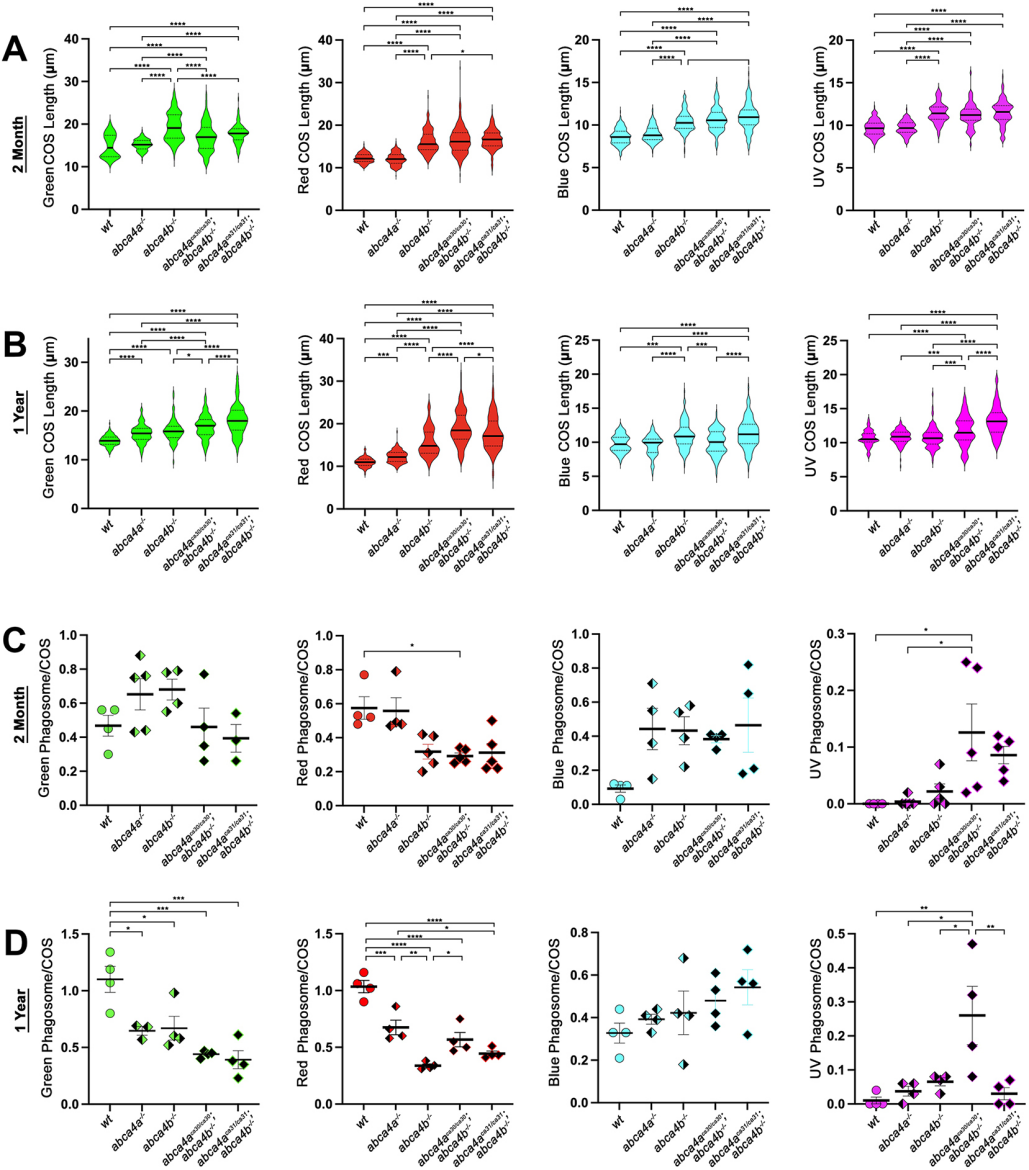
**Quantification of COS length and number of phagosomes per COS in *abca4* mutant fish aged 2 month or 1 year.** (A,B) Lengths measurements of individual cone outer segment (COS) subtypes in retinas obtained from 2-month-old (A) and 1-year-old (B) wild-type (*wt*) or *abca4a^ca30/ca30^* (*abca4a^−/−^*), *abca4b^ca33/ca33^* (*abca4b^−/−^*), *abca4a^ca30/ca30^*;*abca4b^ca33/ca33^* (*abca4a^ca30/ca30^*;*abca4a^−/−^*) and *abca4a^ca31/ca31^*;*abca4b^ca33/ca33^* (*abca4a^ca31/ca31^*;*abca4b^−/−^*) mutant zebrafish. Data for each genotype are combined measurements from at least three eyes obtained from individual fish (individual measurements are provided in Fig. S2). Solid horizontal lines represent the mean; dashed horizontal lines indicate quartiles. (C,D) Numbers of shed COS phagosomes were normalized to the number of COS for each cone type in 2-month-old (C) or 1-year-old (D) wild-type (*wt*) or *abca4a^ca30/ca30^*, *abca4b*^−*/*−^, *abca4a^ca30/ca30^*;*abca4b^ca33/ca33^* and *abca4a^ca31/ca31^*;*abca4b^ca33/ca33^* mutant fish. Each symbol represents data from an image from an eye from an individual fish; horizontal line represents the mean and vertical error bars indicate the s.e.m. *P<0.05, **P<0.01, ***P<0.001, ****P<0.0001 (one-way ANOVA, significance by Tukey's multiple comparisons test).

Because we observed that many COS appeared longer than normal in *abca4* mutants, we sought to quantify COS length in young (2-month-old) and middle-aged (1-year-old) *wt*, and *abca4a^−/−^*, *abca4b^−/−^* and *abca4a^−/−^*;*abca4b^−/−^* double mutant fish (Fig. 5A,B). Plots of COS lengths are shown as aggregated measurements from at least three individuals per genotype, comprising measurements from individual retina samples (Fig. S2). In either age group, all COS subtypes were significantly longer in *abca4b^ca33/ca33^* and *abca4a^−/−^*;*abca4b^−/−^* double mutants compared to those in *wt* fish (Fig. 5A,B). Green COS were the longest COS subtype, followed by red COS. We also found that in 1-year-old fish, both subtypes were significantly longer in *abca4a^−/−^* single mutants compared to those in *wt* fish (Fig. 5B). Moreover, we not only observed longer COS in *abca4* mutants but that variability in COS lengths was generally increased (Fig. 5A,B).

Photoreceptor outer segments are continuously renewed by the combined processes of growth at the base (i.e. by addition of new membranous disks and resident membrane proteins) and shedding (or loss) of outer segment material from the tip that forms the phagosomes within the RPE (Young, 1967; Young and Bok, 1969; Anderson et al., 1978); thus, outer segments are kept fresh and functional for a lifetime. Because COS were longer in *abca4* mutants and because the number of phagosomes appear to be altered in *abca4* mutants, we sought to quantify phagosomes in retinas of *wt* and *abca4* mutant fish (Fig. 5C,5D). We counted phagosomes in confocal images of at least three individual retinas and normalized their number to that of COS in the respective images. At age 2 months, the number of red phagosomes was significantly reduced in the retina of *abca4a^ca30/ca30^*;*abca4b^ca33/ca33^* double mutants compared to that of *wt* fish*,* while the number of UV phagosomes was significantly increased in the retina of *abca4a^ca30/ca30^*;*abca4b^ca33/ca3^* double mutant compared to *wt* fish (Fig. 5C). At age 1 year, the number of green and red phagosomes was significantly reduced in all retinas of *abca4* mutants compared to those of *wt* fish*,* while the number of UV phagosomes was significantly increased in the retina of *abca4a^ca30/ca30^*;*abca4b^ca33/ca3^* double mutants compared to that of *wt* fish (Fig. 5D).

### Photoreceptors in very young and very old *abca4* mutant zebrafish

**Fig. 6.**
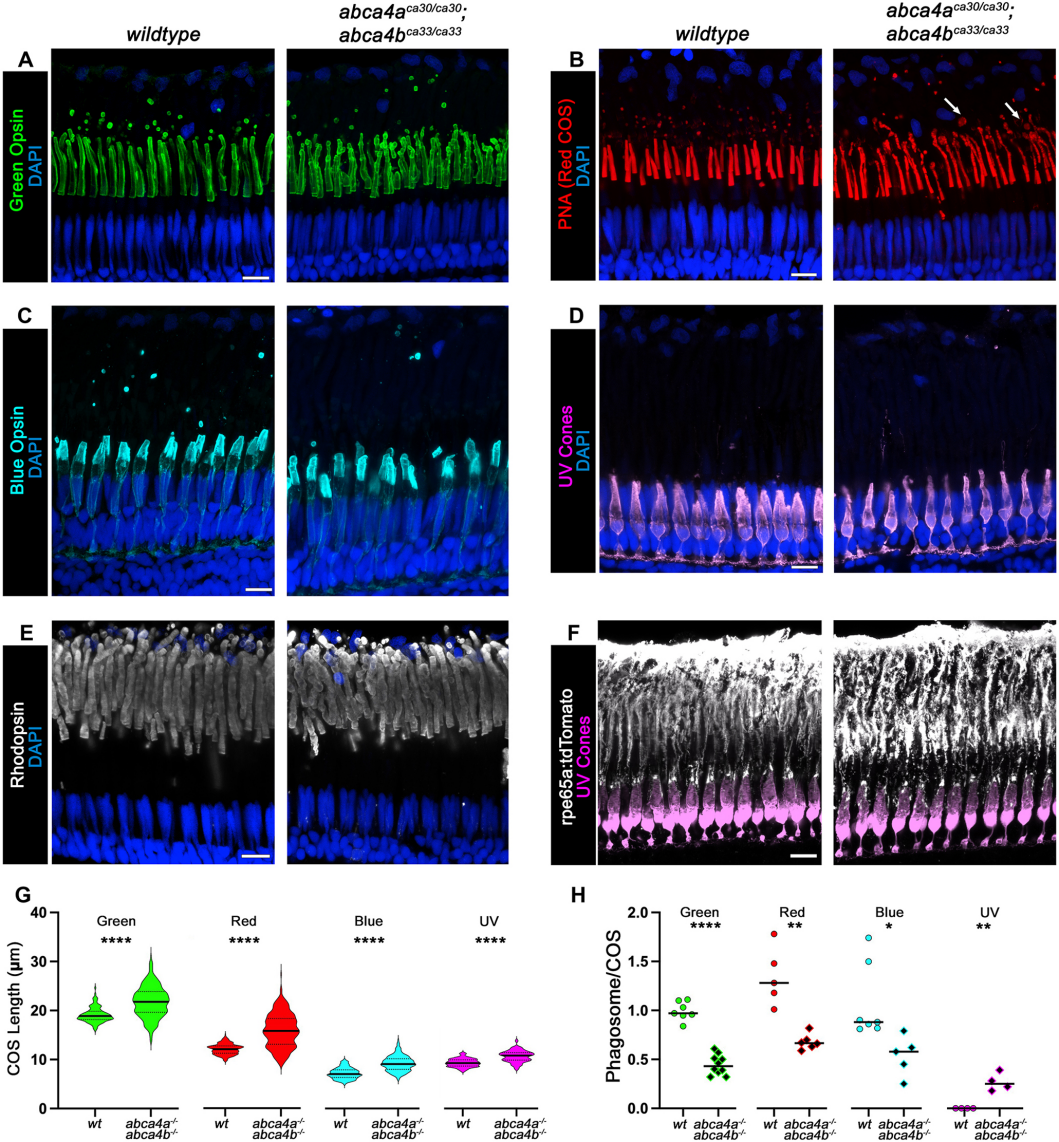
**Photoreceptors, RPE and phagosomes in retina of young juvenile wild-type or *abca4* double mutant zebrafish.** (A-F) High-resolution confocal *z*-projections of retina obtained from wild-type or double *abca4a^ca30/ca30^*;*abca4b^ca33/ca33^* mutant zebrafish at 5 weeks of age. Images show (A) green cone outer segments (COS) stained for Green Opsin (green, *z*=3.24 µm); (B) red COS labeled with PNA (PNA, *z*=4.5 µm) with distal tip bulges indicated by white arrows; (C) blue COS stained for Blue Opsin (cyan, *z*=4.5 µm); (D) UV cones expressing EGFP (violet, *z*=5 µm); (E) rod outer segments (ROS) stained for rhodopsin (white, *z*=2.7 µm) and (F) retinal pigmented epithelium (RPE) expressing tdTomato labeled for RFP (white, *z*=3.24 µm). Nuclei are shown in blue (DAPI). Scale bars: 10 µm. (G) Quantification of COS length. Solid horizontal lines represent the mean and dashed horizontal lines indicate quartiles. (H) Quantification of shed phagosomes per COS. Solid horizontal line represents the mean. *P<0.05; **P<0.01; ****P<0.0001 (Welch's *t*-test).

We next sought to examine COS (as described for Figs 3 and 4) in very young (5-week-old) *abca4* mutant fish, i.e. at an age when outer segments of cones are still elongate to reach a mature steady-state length. For that, we labeled retinas of *wt* and *abca4a^−/−^*;*abca4b^−/−^* double mutant zebrafish aged 5 weeks (young juveniles). Visualization by higher resolution confocal microscopy (Nikon AX R NSPARC confocal) revealed that − even at this young age − COS of mutant fish are already abnormal (Fig. 6). In *wt* retina, green COS are straight and uniformly stained for Green Opsin, but in *abca4a^−/−^*;*abca4b^−/−^* double mutant retina, some green COS exhibit pockets of more-intense Green Opsin staining, suggesting buckling of the COS structure (Fig. 6A). In *wt* retina, the slightly shorter red COS are uniformly stained for PNA, but in *abca4a^−/−^*;*abca4b^−/−^* double mutant retina, red COS are much longer, with regions of discontinuous PNA staining; moreover, some COS have distinct, large distal tip bulges (Fig. 6B and Fig. S5B). In *wt* retina, the short blue COS stained for Blue Opsin, are broad, tapering to flat tops, whereas in *abca4a^−/−^*;*abca4b^−/−^* double mutant retina they are tubular and most have rounded tops (Fig. 6C). In *wt* retina, the most basal UV COS are cone shaped and extend to the top of the outer nuclear layer (the nuclei of rods and cones), but in *abca4a^−/−^*;*abca4b^−/−^* double mutant retina, they are thin and tubular, and extend well beyond the outer nuclear layer (Fig. 6D). Fewer green, red, blue phagosomes are apparent in *abca4a^−/−^*;*abca4b^−/−^* double mutant retina compared to *wt*, while there are more UV phagosomes in *abca4a^−/−^*;*abca4b^−/−^* double mutant retina (near the RPE cell surface) compared to *wt* (Fig. 6A-D). ROS structures in *wt* and *abca4a^−/−^*;*abca4b^−/−^* double mutant appeared to be similar (Fig. 6E). RPE morphology revealed by expression of *rpe65a:tdTomato* was similar between *wt* and *abca4a^−/−^*;*abca4b^−/−^* double mutant retina, including the very long RPE processes/microvilli that cup the UV COS (Fig. 6F). High-contrast images of photoreceptor outer segments of *wt* and double mutants at 5 weeks are provided in the supplemental material (Fig. S3).

We measured the length of COS subtypes in the retina of 5-week-old zebrafish and found that all COS subtypes are significantly longer in *abca4a^−/−^*;*abca4b^−/−^* double mutants compared to *wt* (Fig. 6G). Plots of COS length are the aggregated measurements from at least three individuals per genotype, measurements from individual retina samples are provided in the supplemental material (Fig. S4). We counted phagosomes and normalized them to the number of COS in the image, and found that green, red and blue phagosomes were significantly reduced in *abca4a^−/−^*;*abca4b^−/−^* double mutants compared to *wt*, while the number of UV phagosomes was increased in *abca4a^−/−^*;*abca4b^−/−^* double mutants (Fig. 6H).

Finally, we examined COS, ROS and RPE in retinas of aged – i.e. near the end of the typical zebrafish lifespan – 2-year-old *wt*, *abca4b^−/−^* and *abca4a^−/−^*;*abca4b^−/−^* double mutants (Fig. S5). Qualitatively, the morphology of COS, ROS and RPE is similar to that observed for younger fish, and ROS phagosomes were present in the RPE in both *wt* and *abca4a^−/−^*;*abca4b^−/−^* double mutant retinas (Fig. S5G).

### In *abca4^−/−^* double mutants the structural integrity of COS is disrupted

**Fig. 7.**
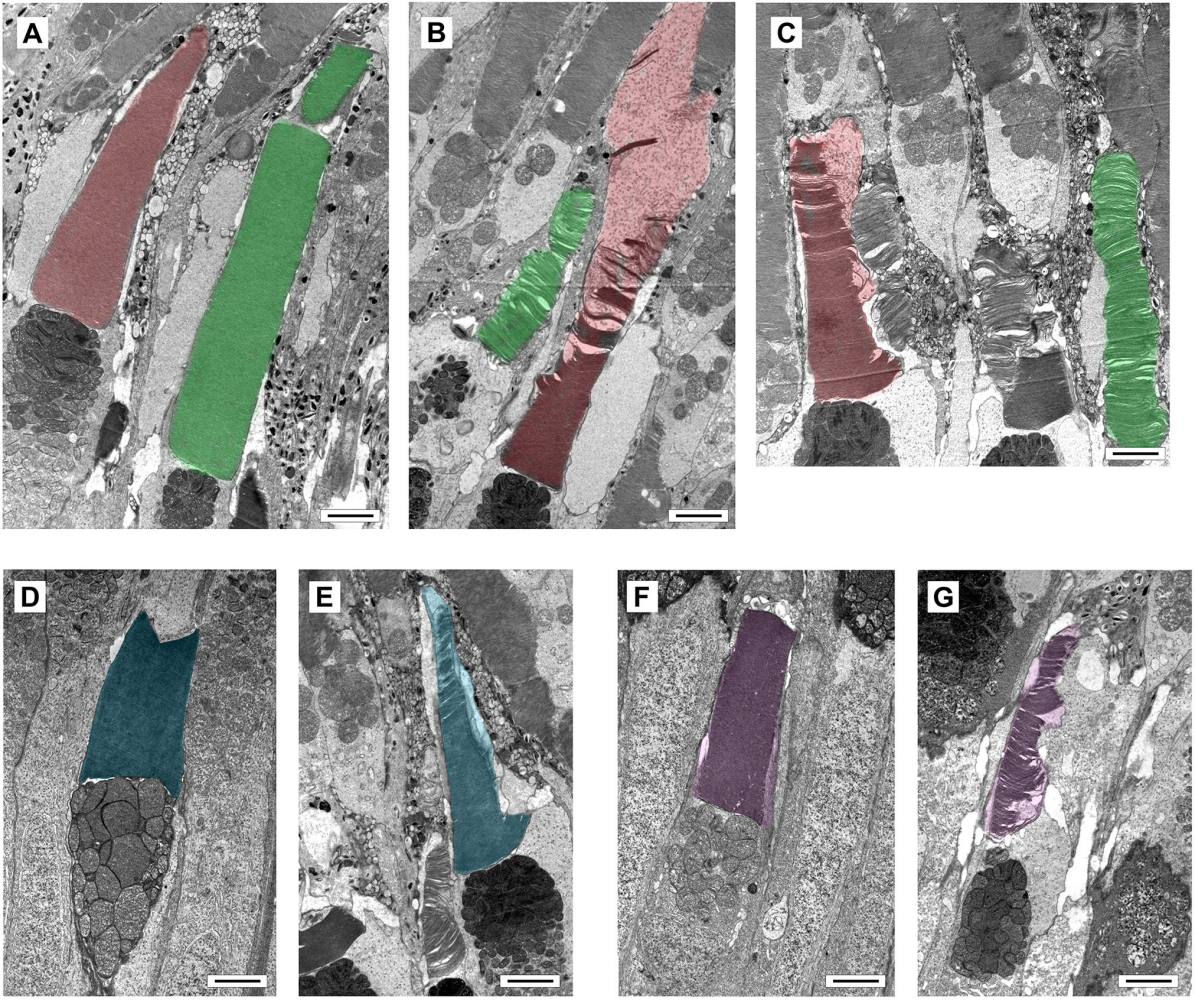
**Ultrastructure of cone outer segments in retinas of wild-type and *abca4* double mutant zebrafish.** (A-G) TEM images of pseudo-colored COS obtained from 4-month-old *wild-type* or *abca4*^−/−^ (*abca4a^ca31/ca31^*;*abca4b^ca33/ca33^*) double mutant zebrafish. Green and Red COS in retina of *wild-type* (A) or *abca4*^−*/*−^ (B,C) fish. Blue COS in retina of *wild-type* (D) or *abca4*^−*/*−^ (E) fish. UV COS in retina of *wild-type* (F) or *abca4*^−*/*−^ (G) fish. Scale bars: 2 µm.

To better understand the role Abca4 has in COS, we used transmission electron microscopy (TEM) to examine the COS structure within retinas of *wt* and *abca4a^−/−^*;*abca4b^−/−^* double mutant zebrafish at 4 months of age. Obtained images revealed that COS disk structure, packing and alignment are severely disrupted in all cone subtypes (Fig. 7). A *wt* green COS shows tightly packed and well-aligned disks along the entire length, with a phagosome present at the tip (Fig. 7A). A *wt* red COS showed tightly packed, aligned disks that taper toward the tip (Fig. 7A). Examples of *abca4a^−/−^*;*abca4b^−/−^* double mutant green COS showed that disks are poorly packed and misaligned (Fig. 7B,C). Examples of *abca4a^−/−^*;*abca4b^−/−^* double mutant red COS showed that disks are tightly packed towards the outer segment base but that, further away, disks become disorganized and appear to disintegrate into vesicles toward the tip (Fig. 7B,C). A *wt* blue COS showed tightly packed aligned disks (Fig. 7D), while those in the *abca4a^−/−^*;*abca4b^−/−^* double mutant were narrower, densely packed at the base but less well packed distally, with vesicles present in the tip (Fig. 7E). A *wt* UV COS showed tightly packed aligned disks (Fig. 7F), while those in the *abca4a^−/−^*;*abca4b^−/−^* double mutant are less well packed, having varying diameter along the length of the outer segment (Fig. 7G). Higher-resolution images of COS are provided in Fig. S6. We also examined ROS in *abca4a^−/−^*;*abca4b^−/−^* double mutants and showed that their structure is much less affected than COS, showing that disks are tightly packed along most of the ROS length but less well packed towards the tip (Fig. S7). The mitochondria of *abca4a^−/−^*;*abca4b^−/−^* double mutant rods appeared swollen, indicating cell stress (Fig. S7F).

Because the RPE has been suggested to play a role in STGD (Molday et al., 2022), we examined the RPE ultrastructure in retinas of *wt* and *abca4a^−/−^*;*abca4b^−/−^* double mutant fish at ages 4 months and 1 year. At 4 months, the RPE of *wt* and *abca4^−/−^* double mutant fish retinas were similar, and mitochondria appeared healthy in the mutant RPE (Fig. S8). However, at 1 year of age (Fig. S9), more outer segment debris was apparent in the RPE of mutant fish, and the number of lipofuscin droplets was increased compared to those of *wt* RPE, in which none were observed (Fig. S9).

### Cone outer segments constitutively display externalized phosphatidylserine

The molecular mechanisms that control outer segment shedding and phagocytosis by RPE are poorly understood. The externalization of the phospholipid phosphatidylserine (PS) serves as a well-described ‘eat-me’ signal for phagocytosis (reviewed by Segawa and Nagata, 2015). Using an Annexin 5 probe that labels PS with high affinity, externalized PS has been observed at ROS tips in acutely dissected, live mouse retina and has been suggested to play a role in the shedding process (Ruggiero et al., 2012). We sought to examine externalized PS in zebrafish and its potential role in outer segment phagocytosis in *abca4a^−/−^*;*abca4b^−/−^* mutants. Labeling of externalized PS requires intact plasma membrane integrity because if membrane integrity is disrupted the PS-probe will label PS on the inner membrane leaflet. To label externalized PS *in vivo*, we created a doxycycline (DOX)-inducible, secreted V5-tagged Annexin 5 PS sensor (*TRE:secA5^V5^*) and injected this plasmid into a pan-cone TetOn driver line (Fig. S10A) to create genetic mosaics, in which a small number of cones would secrete secA5^V5^ into the photoreceptor layer. At 10 months of age, we induced secA5^V5^ expression by DOX treatment for 2 days. We observed no labeling of ROS tips by secA5^V5^; however, COS as well as COS phagosomes were extensively labeled (Fig. S10B). Because binding of Annexin 5 to PS is calcium sensitive (Tait and Gibson, 1992), and local extracellular calcium potentially impacts secA5^V5^ binding to ROS tips *in vivo*, we created a second genetically encoded PS sensor that is calcium insensitive, i.e. secLacC2^V5^ (Yeung et al., 2008). We injected this *TRE:secLacC2^V5^* construct into pan-cone TetOn driver line embryos and treated adults (mosaic for integrated *TRE:secLacC2*) with DOX for 3 days. Although COS labeling was similar to that observed for secA5^V5^ − with additional labeling at the base of ROS, forming one to two stripes − ROS tip labeling was still not observed (Fig. S10C), and no staining for V5-tagged Annexin 5 was observed in non-injected fish that had been DOX treated (Fig. S10D-G). We considered that ROS tip labeling might require higher and longer expression of secLacC2^V5^ and DOX-treated fish for 14 days. Still, no ROS tip labeling was observed in response but, rather, a striking pattern of secLacC2^V5^ stripes in ROS appeared, with the number of stripes (12-13) being close to the number of days of DOX-treatment (Fig. S10H).

**Fig. 8.**
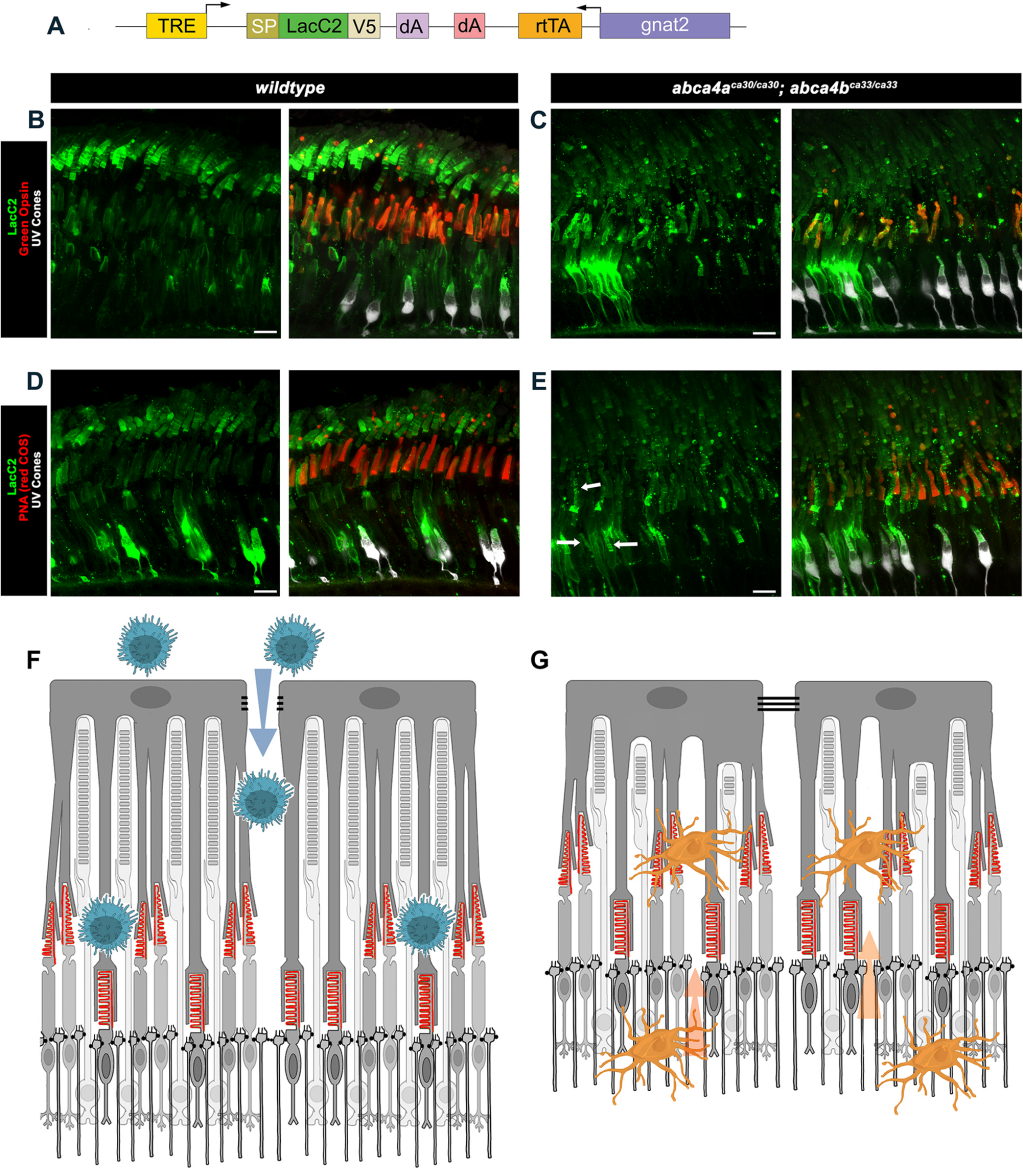
***In vivo* labeling of externalized phosphatidylserine.** (A) Schematic of the doxycycline (DOX)-responsive V5-epitope-tagged LacC2^V5^ phosphatidylserine (PS)-sensitive DNA construct that was injected into single-cell wild-type or *abca4^ca30/ca30^;abca4b^ca33/ca33^* embryos. TRE, tetracycline response element; SP, signal peptide; gnat2, *gnat2* promoter sequence; rtTA, reverse tetracycline-controlled transactivator; dA, polyA sequence. (B-E) Confocal *z*-projection (*z*=4.4 µm) images showing the photoreceptor layer in 4-month-old wild-type (B,D) or *abca4^ca30/ca30^;abca4b^ca33/ca33^* mutant (C,E) fish that had been treated with doxycycline for 7 days, and co-labeled for V5 (LacC2, green) and Green Opsin (red) or V5 (LacC2, green) and PNA (red) as indicated. EGFP-expressing UV cones are shown in white. (A-E) Very brightly labeled cells have incorporated the transgene, and make and secrete LacC2 protein. In the wild-type retina, LacC2 labeling smoothly coats the surface of COS and in ROS forms distinct stripes beginning at the ROS base. In the *abca4a*^−/−^;*abca4b*^−/−^ double mutant retina, LacC2 labeling in COS is disrupted, with the appearance of puncti and stripes of LacC2 labeling (examples indicated by arrows in E), and ROS labeling appears less intense. Scale bars: 10 µm. (F) Schematic of how loss of RPE tight-junction (TJ) integrity in retinal degeneration diseases, like AMD, might allow macrophage (blue) infiltration, and how recognition of COS-externalized PS (indicated in red) could result in COS attack. (G) Schematic of how disruption of the outer limiting membrane (OLM) − which is formed by adherens junctions between Müller glia and photoreceptors, in retinal rod degeneration diseases, like retinitis pigmentosa − could allow microglial (orange) infiltration and recognition of the phagocytic ‘eat-me’ signal of externalized PS on COS, resulting in COS attack.

Because it appeared that *wt* COS constitutively display the phagocytic ‘eat-me’ signal of externalized PS, and because COS phagocytosis was disrupted in *abca4a^−/−^*;*abca4b^−/−^* mutants, we sought to visualize externalized PS on COS in the retina of these mutants. For this, we created another construct that includes both sequences of the pan-cone TetOn driver *gnat2:rtTA* and of the DOX-response gene *TRE:secLacC2^V5^* (Fig. 8A). We injected this construct into *wt* and *abca4a^−/−^*;*abca4b^−/−^* double mutant embryos, and then DOX-treated mosaic adults for 7 days. In *wt* retina, secLacC2^V5^ smoothly labeled entire COS and formed stripes in ROS; COS PS-labeling was confirmed by double-labeling for Green Opsin and PNA (Fig. 8B,D). In retina *abca4a^−/−^*;*abca4b^−/−^* double mutants, secLacC2^V5^-labeled COS but, in many cases, labeling was in stripes (Fig. 8C,E, see arrows in panel E) and more punctate and the ROS stripes seem fainter, although this method is not quantitative (Fig. 8C,E). Additional examples of secLacC2^V5^ labeling are provided in Fig. S11.

## DISCUSSION

In this study, we sought to create a model of STGD in the cone-rich retina of zebrafish that would reveal mechanisms of photoreceptor dysfunction and lead to a better understanding of disease processes to help accelerate progress towards improving treatments to slow or prevent vision loss. By using CRISPR/Cas9, we generated null mutants in the two zebrafish paralogues *abca4a* and *abca4b* of human *ABCA4*, and examined single mutants in each paralogue as well as in *abca4a;abca4b* double mutants. Abca4 antibody labeling of retina from these single and double mutants indicated that *abca4a* and *abca4b* are both null mutants because we observed no Abca4 labeling in *abca4a;abca4b* double mutants and found that most Abca4 protein in zebrafish is encoded by *abca4b*. Previously, the localization of Abca4 protein has been reported in ROS, COS and RPE of various species, including mouse, dog and human (Sun and Nathans, 1997; Mäkeläinen et al., 2019; Molday et al., 2000; Lenis et al., 2018). Here, we showed that staining of zebrafish retina for Abca4 protein manifests as a ladder-like pattern in ROS, with higher Abca4 levels at disk rims, whereas in COS, Abca4 presents as a thick, jagged stripe extending throughout the length of the COS, with some Abca4 colocalizing with opsin and Abca4 puncta visible on COS phagosomes. We did not observe Abca4 in RPE cell bodies, but the sprinkling of Abca4 in the COS region might be due to RPE microvillar labeling (Fig. S1).

Previous studies using *Abca4*-knock out and knock-in mice have revealed only modest reduction in rod function and mild degeneration of rods (Weng et al., 1999; Zhang et al., 2015; Molday et al., 2018). Also, a pair of Labrador retriever siblings has been identified as being homozygous for a single nucleotide insertion within the *Abca4* gene, predicted to truncate the protein at the beginning of nucleotide binding domain two (ND2) (Mäkeläinen et al., 2019). Retinas had been examined when the dogs were 12 years old. At this time, nearly all cones were absent, preventing assessment of COS morphology, while most rods had remained albeit with reduced function compared to age-matched controls. The cone-rich retina of zebrafish provides an accessible opportunity to investigate how loss of Abca4 function affects cone structure and function over the lifespan of these fish.

All COS subtypes in zebrafish *abca4* double mutants were morphologically abnormal from the youngest age examined, with mutant green COS appearing crumpled and/or buckled, PNA labeling of mutant red COS discontinuous and a large distal tip bulge frequently observed. Moreover, mutant blue and UV COS were thinner and more tubular in mutant than in *wt* fish. All COS subtypes were longer than normal. This is an unexpected finding since a general feature of photoreceptor degenerative diseases − primarily studied in rod degenerative models − is the progressive shortening of outer segments that precedes photoreceptor death (Heckenlively et al., 1995; Hawes et al., 2000; Hong et al., 2000; Gao et al., 2002; Collin et al., 2005). A view of COS ultrastructure in *abca4*^−/−^;*abca4*^−/−^ revealed the disk and membrane structural defects (Fig. 7, Fig. S6) underlying the morphology abnormalities observed by light microscopy (Figs 2, 3, 4 and 6, Fig. S3). Disk packing and morphology were severely disrupted in all *abca4^−/−^* COS subtypes, with disks appearing to disintegrate into vesicles, and disintegration becoming more pronounced towards the distal tip. In contrast, ROS morphology appeared largely normal in *abca4^−/−^* mutants, with minor loss of disk density and/or packing towards the tip; therefore, it is possible that zebrafish do not live long enough for more-severe rod defects to present. Our analyses of wild-type and *abca4^−/−^* mutant zebrafish were performed in the *albino* (*alb^−/−^*) background, which disrupts the *slc45a2* gene that encodes a proton transporter in melanosomes (Dooley et al., 2013). Although loss of *slc45a* does not affect contrast sensitivity in zebrafish (Dooley et al., 2013), we have not ruled out that the *albino* mutation might enhance of the *abca4^−/−^* phenotype.

Outer segments, highly modified primary cilia, of rods and cones undergo a continual process of renewal during the combined daily processes of proximal growth and distal shedding, and these processes are balanced in mature photoreceptors to maintain constant length throughout the lifespan. Outer segment renewal has been studied almost exclusively in rods and, while molecular mechanisms controlling the process are largely unknown, the photoperiod has been repeatedly shown to be a principal factor that stimulates both ROS and COS tip shedding, with shedding occurring for both types within hours of light onset (Young, 1977, 1978; Fisher et al., 1983). While we observed reduced numbers of red, green and blue phagosomes in *abca4^−/−^* mutants, UV phagosome numbers were increased. We suspect this observation reflects that UV COS shedding is not entrained to the same light cycle as those COS that respond to visible light spectra. Moreover, the presence of UV phagosomes in mutants suggests that, at the time of retinal fixation, the RPE is still digesting shed UV COS material. Reduced cone functional response to light could contribute to the diminished COS shedding and phagocytosis by the RPE we observed in in *abca4^−/−^* mutants.

What could be the role(s) of Abca4 in contributing to COS structure and tip shedding/phagocytosis by the RPE? Abca4, a member of the ABC cassette transporter superfamily of proteins, consists of twelve transmembrane domains, two nucleotide-binding domains and two large glycosylated ectodomains (reviewed by Molday et al., 2022). Abca4 has been shown to have transporter activity for N-retinylidene-phosphatidylethanolamine (NRPE), the reversible covalent adduct of all-*trans* retinal (ATR) and phosphatidylethanolamine (PE), across the lipid bilayer and, thus, has been proposed to be a first step towards regenerating 11-*cis* retinal (in the RPE) following its isomerization to ATR by light absorption in rhodopsin (reviewed by Molday et al., 2022). One principal structural difference between ROS and COS is that, in ROS, the discrete stacked membranous disks that contain the phototransduction machinery are entirely enclosed by the plasma membrane, whereas COS disks comprise a single continuous lamellar membrane that is open to the extracellular space, i.e. open disks (Eckmiller, 1987; reviewed by Goldberg et al., 2016). The large Abca4 ectodomains have been predicted to reside on the lumen side of ROS disks and proposed to contribute to ABCA4 localization at the hairpin rim of ROS disks (Sun and Nathans, 1997; reviewed by Molday et al., 2022). In COS, the large glycosylated ectodomains of Abca4 would be extracellular. This feature presents the possibility that these domains contribute to COS lamellar disk formation, disk alignment and disk stability, or even interact with other extracellular proteins or the extracellular matrix to support COS structural integrity.

In the absence of ABCA4 function, it has been postulated that NRPE accumulates in ROS and phagocytosis of the NRPE-ladened ROS material becomes increasingly toxic to the RPE and, consequently, photoreceptors degenerate owing to lost RPE support (Radu et al., 2004; reviewed by Tsybovsky et al., 2010). Lipofuscin accumulation has been observed in patients diagnosed with STGD (Mata et al., 2000; Burke et al., 2014) and in mouse STGD models (Weng et al., 1999; Mata et al., 2000), although its significance is uncertain. We found the ultrastructure of RPE in retina of *abca4^−/−^* zebrafish is similar to that of *wt* at 4 months, but, by the age of 1 year, RPE abnormalities are apparent in *abca4^−/−^* mutant, with more outer segment debris and abundant levels of lipofuscin present, thereby indicating that disease features progress with age.

A key signal for phagocytosis is the display of externalized PS on cell membranes (reviewed by Segawa and Nagata, 2015). We showed here that, by using two *in-vivo* probes, *wt* COS constitutively display externalized PS, an ‘eat-me’ signal, and this pattern of externalized PS is disrupted in *abca4* mutant COS. Whether RPE uses externalized PS to recognize COS for phagocytosis remains unknown but if it is an important cue then the disorganized externalized PS observed in *abca4* mutant COS could impact RPE recognition and contribute to the observed phagocytic deficiency.

The photoreceptor outer segment region is generally considered to be an acellular compartment, with RPE tight junctions on one side and the outer limiting membrane (OLM), formed by adherens junctions between photoreceptors and Müller glia, on the other side (Williams et al., 1990). Thus, together, these structures effectively block entrance of immune cells that otherwise would encounter externalized PS on COS. We propose models of how externalized PS on COS, if conserved in human COS, could contribute to cone degeneration in retinal degeneration diseases. One feature of age-related macular degeneration (AMD) is RPE atrophy and, thus, loss of blood−retinal barrier integrity (reviewed by Somasundaran et al., 2020). Loss of RPE tight junction barrier function can allow macrophages to enter the outer segment region and encounter COS that present the ‘eat-me’ signal of externalized PS (Fig. 8F). Retinitis pigmentosa is an inherited retinal degeneration disease that primarily causes degeneration of rod photoreceptors but, secondarily, also that of cone photoreceptors (reviewed by Verbakel et al., 2018). Most mutations causing retinitis pigmentosa are in genes that are largely or exclusively expressed in rods; the cause of the secondary cone degeneration is uncertain, although several mechanisms have been proposed (reviewed by Campochiaro and Mir, 2018; Song et al., 2023). When OLM barrier function is disrupted by rod degeneration this could open an avenue of microglia invasion into the outer segment region, where they would encounter externalized PS on COS and initiate pathogenic phagocytic activity (Fig. 8G). We did not identify the cell type of those nuclei observed in the photoreceptor outer segment region that are rare in *wt* retina, where they are usually adjacent/near the RPE apical surface. These nuclei are more numerous in *abca4* mutants and spread throughout the outer segment region (see Fig. 3B,C, yellow arrows). If these cells represent a resident population of quiescent microglia near RPE in the healthy retina, perhaps surveilling for microbe invasion across tight junctions, they might be activated by inflammatory molecules, including those strongly associated with AMD (reviewed by Pan et al., 2023; de Jong et al., 2023), to attack COS that present the ‘eat-me’ signal?

The quantifiable cone photoreceptor phenotype observed in the zebrafish *abca4* mutants provides the foundation to examine the two separatable, potential Abca4 protein mechanisms for supporting COS structure, phagocytosis and function: (1) structural and, (2) transporter. By using genome engineering, each feature of Abca4 can be disrupted independently (ectodomains versus catalytic activity), and subsequent effects on COS can be examined and quantified, which is an important step towards understanding the cause of cone degeneration in STGD and developing therapeutic interventions that target the molecular basis of disease. Given the role of Abca4 in COS structural integrity as shown in our study, therapies aimed solely at treating the transporter deficiency may be insufficient to restore cone function in patients diagnosed with STGD.

Development of sensitive flicker/flash electroretinography (ERG) methods to isolate and quantify cone function in zebrafish is needed to complement and add to the findings presented here. While the retina of zebrafish provides the opportunity to study cones, the robust regenerative capacity of zebrafish can obscure the extent and blunt the impact of photoreceptor degeneration upon retinal examination by microscopy and function by ERG, especially when degeneration is slow (reviewed by Lenkowski and Raymond, 2014). Thus, we did not attempt to quantify photoreceptor numbers or degeneration in this study, although reduced COS subtypes are apparent in many of the images presented (Fig. S5). The impact of light exposure on progression in patients diagnosed with STGD remains uncertain (Teussink et al., 2015), and this zebrafish STGD model provides the opportunity to examine whether and how light − i.e. duration and specific wavelengths − impacts photoreceptor structure and function, and accumulation of lipofuscin.

## MATERIALS AND METHODS

### Animal maintenance

This study was carried out in strict accordance with the recommendations in the Guide for the Care and Use of Laboratory Animals of the National Institutes of Health; the protocol was approved by the University of Massachusetts Amherst Institutional Animal Care and Use Committee. All fish lines were maintained in a mixed background of *AB*; *albino^b4/b4^* (Dooley et al., 2013) and maintained on a 12:12 light/dark cycle. The size and length of young juveniles (5 weeks of age) was not significantly different between genotypes (mean length, snout to base of the tail, ∼11 mm, standard error =0.37 mm). To induce TetOn gene expression, fish were exposed to 5 mg/ml doxycycline (DOX)/day in non-circulating fish system water and fed each day during the 4-h period of recirculating water. Fish were euthanized with Tricaine-S (Syndel, Ferndale WA, USA) prior to tissue collection.

### Genome engineering

#### CRISPR/Cas9

Guide/trac RNA was made from filled in oligo templates and capped, tailed Cas9 mRNA was synthesized from linearized pT3TS-nCas9n plasmid (Jao et al., 2013). A mixture of gRNA and Cas9 (50 ng/µl gRNA, 40 ng/µl Cas9 mRNA) was injected into one or two-cell stage embryos. Gene-specific guide sequences were as follows: *abca4a*, CR9 - 5′-GTTGTAGTTGGACACCAGGCC-3′; and *abca4b*, CR10 - 5′-cAATCCCTTGGATCCAAGGC-3′. Recovered and established mutant alleles were genotyped using fin clip PCR followed by HaeIII (*abca4a*) or PspGI (*abca4b*) digestion.

#### Transgenesis

Plasmids from the Tol2kit (Kwan et al., 2007; Villefranc et al., 2007) and from the Tet-On toolkit (Campbell et al., 2012) were used to generate the following transgene constructs. Germ-line transgenic lines were created using the pTol system (Kawakami et al., 2004) by co-injecting the pTol transgene plasmid with *transposase* RNA into one-cell embryos, identifying germ-line founders, and propagating stable, germline transgenic lines (carrying a single transgene copy) that exhibited minimal positional effect variegation.

##### pTol_rpe65aa:tdTomato

A 6.6 kb fragment upstream region from *rpe65a* in the BAC clone CH73 140J24 (BacPac Resources Center, Children's Hospital, Oakland, CA, USA) was cloned into p5′E-MCS to make p5E-RPE65a and recombined by Gateway cloning with pME-tdTomato and pTolDESTR2R4dA using Clonase LRII+ (Invitrogen).

##### pTol_gnat2: rtTA

A 3727-base pair PCR product upstream of the *gnat2* open reading frame gene from AB strain zebrafish gDNA was recombined with pL1L2-rtTA^FLAG^ and pTolDESTR2R4dA.

##### pTol_TRE: SP-A5-V5

The human annexin 5 fragment from pBH-UAS-secA5-YFP (Addgene #32359) was repaired and cloned into pME-MCS; the signal peptide from *crb2b* (Hsu and Jensen, 2010) replaced the *C. elegans* signal peptide sequence at the 5′ end and the V5 epitope tag with a stop codon at the 3′ end. This was recombined with p5E_TRE and pTolDESTR2R4dA.

##### pTol_TRE: SP-LacC2-V5

The bovine mfg-E8 C2 domain was moved from pLact-C2-GFP (Addgene #22852) into pME_MCS, again with *crb2b* signal peptide on the 5′ end and the V5 epitope tag with stop codon on the 3′ end (pME_SP-C2-V5). This was then recombined with p5E_TRE and p3E_polydA into pTolDestCG2.

##### pTol_gnat2:rtTA_TRE: SP-LacC2-V5

The *gnat2* promoter was placed in front of reverse Tet-On transactivator (rtTA) and a HSV poly dA signal and moved to pTolDestCG2 by conventional cloning. The resulting pTol_PC:ToD was recombined with pME_SP-LacC2-V5 and p5E_TRE and p3E_polydA.

### Immunohistochemistry

All shown data were obtained from animals euthanized 2 h (±15 min) after light onset. Tissue was fixed for 1.5 h in 3% paraformaldehyde, embedded in agar and equilibrated in 30% sucrose. Samples were sectioned (thickness=20 μm) using a Leica cryostat. Sections were rehydrated with phosphate-buffered saline (PBS), permeabilized for at least 6 h with 5% goat serum in PBS containing 0.1% Triton X-100+0.1% Tween 20 (Sigma-Aldrich, St. Louis, MO, USA) and incubated overnight at 4°C in primary antibody in PBS containing 0.1% Tween 20 (PBS-Tw). Sections were washed with PBS-Tw, incubated for at least 6 h with the appropriate secondary antibody in PBS-Tw, and washed again with PBS-Tw. Tissue was incubated with additional primary and secondary antibody combinations over subsequent nights and days. Nuclei were labeled by DAPI (0.5 µm/ml ThermoFisher Scientific, Waltham, MA, USA) in the last antibody mixture. Following antibody labeling, samples were mounted with ProLong Gold Antifade Mountant (ThermoFisher Scientific). Staining for Abca4 required heat-induced epitope retrieval (HIER) for immunohistochemistry; for this, tissue was fixed and transferred to slides as described above, slides were heated to 95°C for 20 min in 20 mM Tris pH 9+1 mM EDTA+0.05% Tween-20, then transferred to PBS-Tw before staining for Abca4 was completed as described above.

Primary antibodies used were rabbit anti-RFP and rabbit anti-GFP (Rockland Immunochemicals, Limerick, PA, USA; #600-401-379, #600-401-215); mouse anti-acetylated tubulin (Sigma Aldrich; #T6793); R6-5 mouse IgG_2A_ monoclonal anti-rhodopsin (Röhlich et al., 1989); K-42 mouse IgG3 monoclonal anti-Green Opsin (Röhlich et al., 1989; Campbell and Jensen, 2017); anti-zebrafish Blue Opsin (Kerafast, Boston, MA, USA; #EJH012) (Luo et al., 2004); rabbit polyclonal V5 tag (GeneTex, Irvine, CA, USA; #GTX117997); affinity-purified custom rabbit polyclonal against zebrafish ABCA4 peptide KVEDILKDDETLTA (Pacific Immunology Corp, Ramona, CA, USA).

Secondary antibodies used were Alexa Fluor 488-conjugated goat anti-rabbit (ThermoFisher Scientific; #A11008); Alexa Fluor 546-conjugated goat anti-rabbit (ThermoFisher Scientific; #A11035); Alexa Fluor 647-conjugated goat anti-mouse IgG_2A_ (Jackson ImmunoResearch, West Grove, PA, USA; #115-605-206); Alexa Fluor 647-conjugated goat anti-mouse IgG_3_ (Jackson ImmunoResearch; #115-175-209); Alexa Fluor 647-conjugated goat anti-mouse IgG_2b_ (Jackson ImmunoResearch; #115-605-207); Cy5-conjugated goat anti-rabbit (Jackson ImmunoResearch; #111-176-003). Also used was Alexa Fluor 647-conjugated peanut agglutinin (PNA) (Molecular Probes/ThermoFisher Scientific; #L-32460).

### Light microscopy

Images were generated as *z*-stacks of optical sections with Nyquist sampling using a Nikon A1R-SIMe laser confocal microscope (Figs 2A, 3 and 4) with a 20×/0.75 NA air, 40×/1.3 NA/oil or 60×/ 1.4 NA/oil objective or with a Nikon AX R equipped with NSPARC (Fig. 2B-G′, Figs 6 and 7, Figs S1, S4, S5, S7) using 40×/1.25 NA/SIL and 60×/1.42 NA/oil objective. Images were processed and analyzed using NIS Elements software. Representative images are projections of *z*-sections (thickness) as described in figure legends. Phagosome counts and COS length measurements (using polyline) were taken from 20× images (*x*=300 µm, *z*=8 µm) using Nikon Elements NIS software. Images in Fig. S7 were acquired with a Zeiss LSM 700 using a 20×/0.8 NA/air objective.

### Transmission electron microscopy

Following euthanasia, eyes were removed between 10am-11:15 am, the cornea pierced, and placed in formaldehyde/glutaraldehyde, 2.5% each in 0.1 M sodium cacodylate buffer pH 7.4 (Electron Microscopy Sciences). The following day, fixative was removed and samples washed twice with 0.1 M sodium cacodylate buffer at 4°C, and then incubated with 1% osmium (3 ml 0.1 M sodium cacodylate buffer and 1 ml 4% osmium) for 1 h at room temperature. Samples were washed twice in deionized water, dehydrated through an ethanol series, and washed twice in 100% propylene oxide. Samples were incubated overnight in Spurr's resin/propylene oxide mixture (1:1). Samples were treated three times with fresh Spurr's resin mixture for 1 h, then placed in the partially polymerized and heated for 48 h at 60°C. Thin sections (60-70 nm) were cut and placed on copper support grids and stained with lead citrate (6 min) and uranyl acetate (8 min). Samples were imaged with a Philips CM 10 transmission electron microscope with 80Kv accelerating voltage.

### Data analyses and statistics

Prism 10 software was used for generating graphs, and statistical analyses were performed using unpaired Welch's *t*-test or by one-way ANOVA with significance by Tukey's multiple comparisons test (each column compared with the mean of every other column).

## Supplementary Material

10.1242/dmm.052052_sup1 Supplementary information
