# Supplementary material for: Abca4, mutated in Stargardt disease, is required for structural integrity of cone outer segments
Source: Dis Model Mech. 2025 Jan 10;18(1):DMM052052. doi: 10.1242/dmm.052052 (PMC11744051; doi:10.1242/dmm.052052)
Supplement: Supplementary information [file dmm-18-052052-s1.pdf]

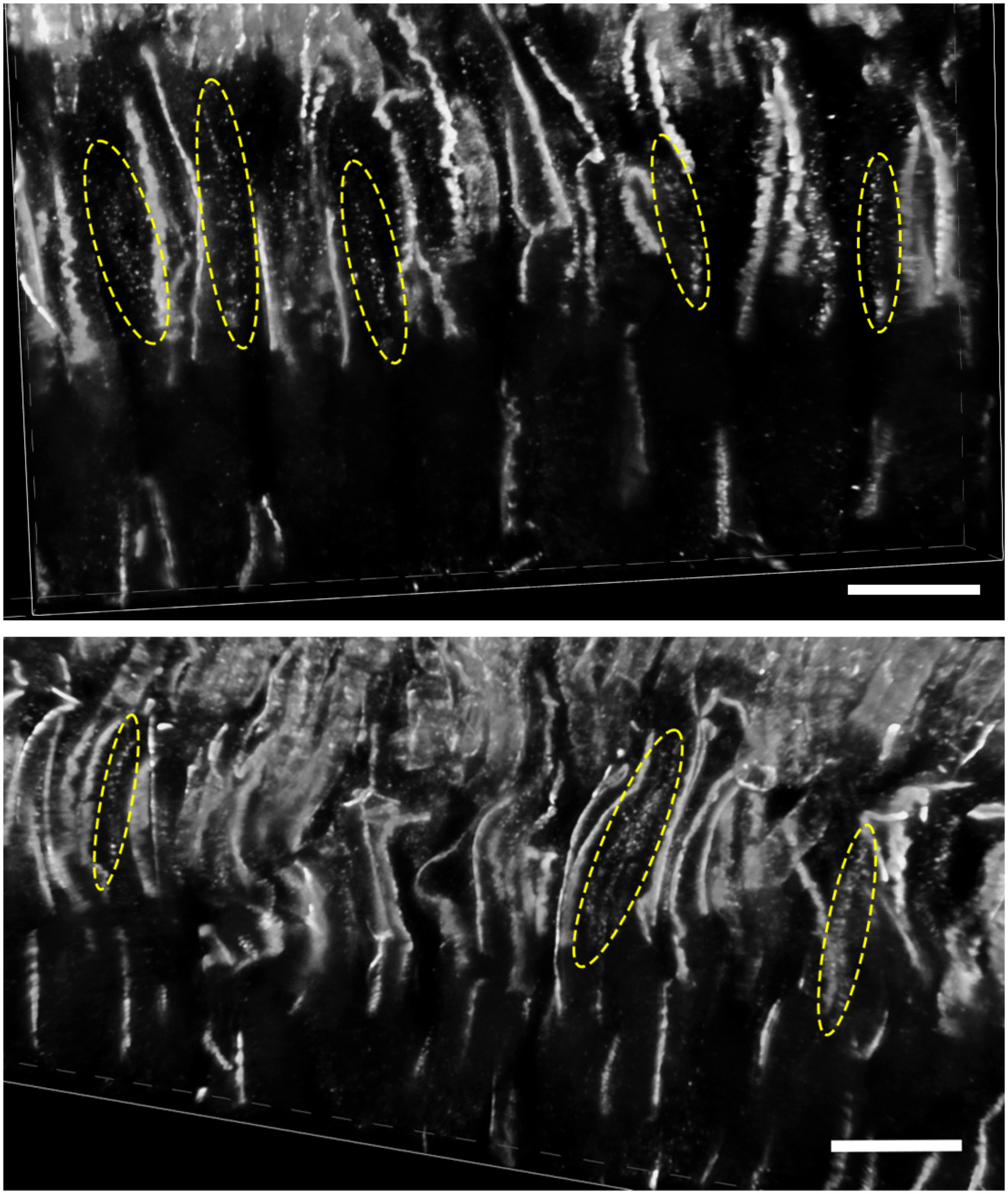

Fig. S1. Volume projections of anti-ABCA4 antibody labeling (white) in the COS region of 4 month *wildtype* retina. Dashed yellow ovals indicate examples of proposed RPE expression. Scale bars, 10 μm.

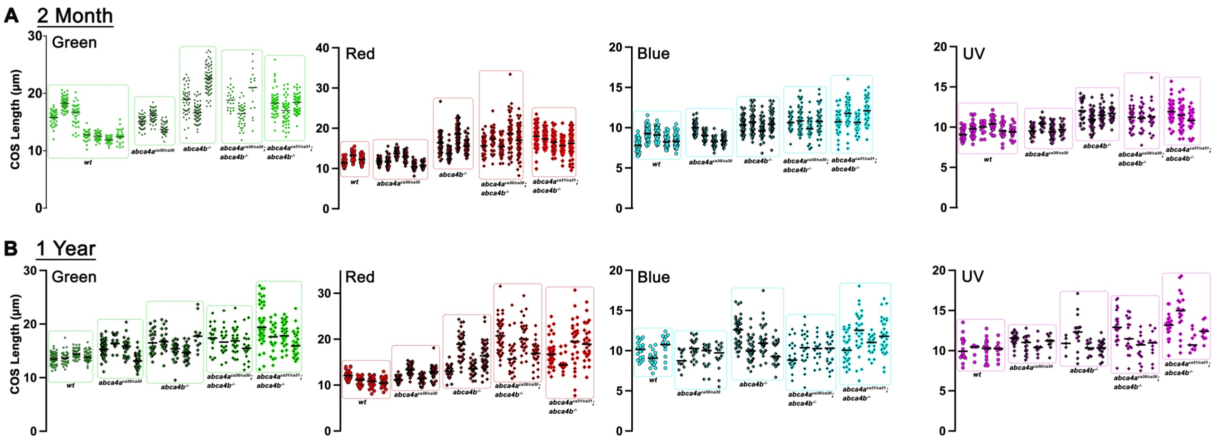

Fig. S2. The individual COS length measurements taken from individual fish used to create Fig. 5A and B. (**A**) 2 months and (**B**) 1 year *wildtype*, *abca4a*$^{-/-}$, *abca4b*$^{-/-}$ and *abca4a*$^{-/-}$; *abca4b*$^{-/-}$ double mutant retina.

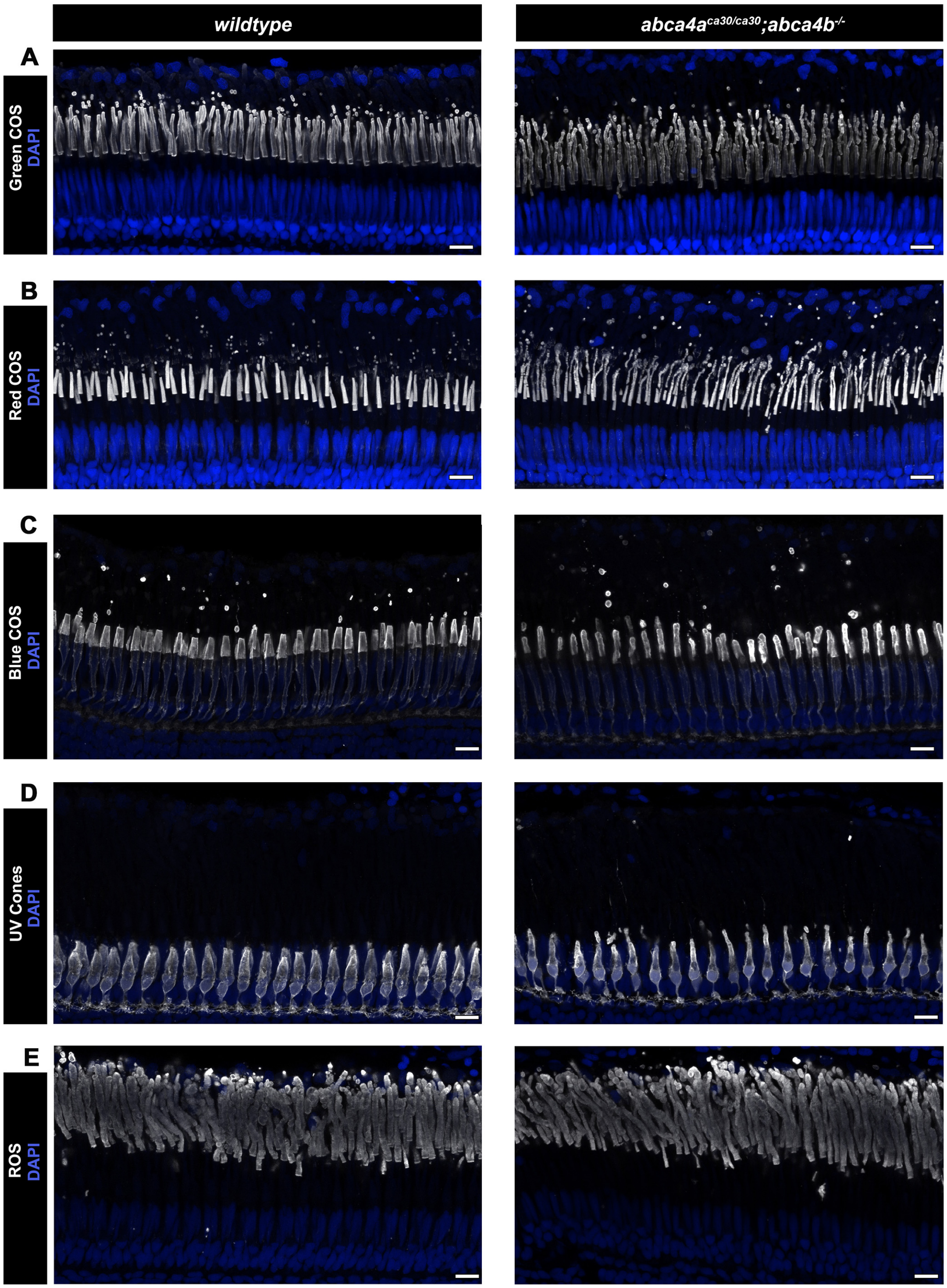

Fig. S3. Additional images of photoreceptors and RPE in 5 week *wild-type* and *abca4a*$^{-/-}$; *abca4b*$^{-/-}$ double mutant retina. High contrast high resolution confocal z-projections of 5 week *wildtype* and *abca4*$^{ca30/ca30}$;*abca4b*$^{ca33/ca33}$ double mutant retina colabeled with DAPI (blue) and (**A**) Green COS labeled with anti-Green Opsin antibodies (white, z = 3.5 μm), (**B**) red COS with PNA (white, z = 5 μm), (**C**) blue COS with anti-Blue Opsin antibodies (white, z = 3.5 μm), (**D**) UV cones expressing EGFP labeled with anti-GFP antibodies (white, z = 4.5 μm), (**E**) ROS with anti-Rhodopsin antibodies (white, z = 2.3 μm). Scale bars, 10 μm.

Disease Models & Mechanisms • Supplementary information

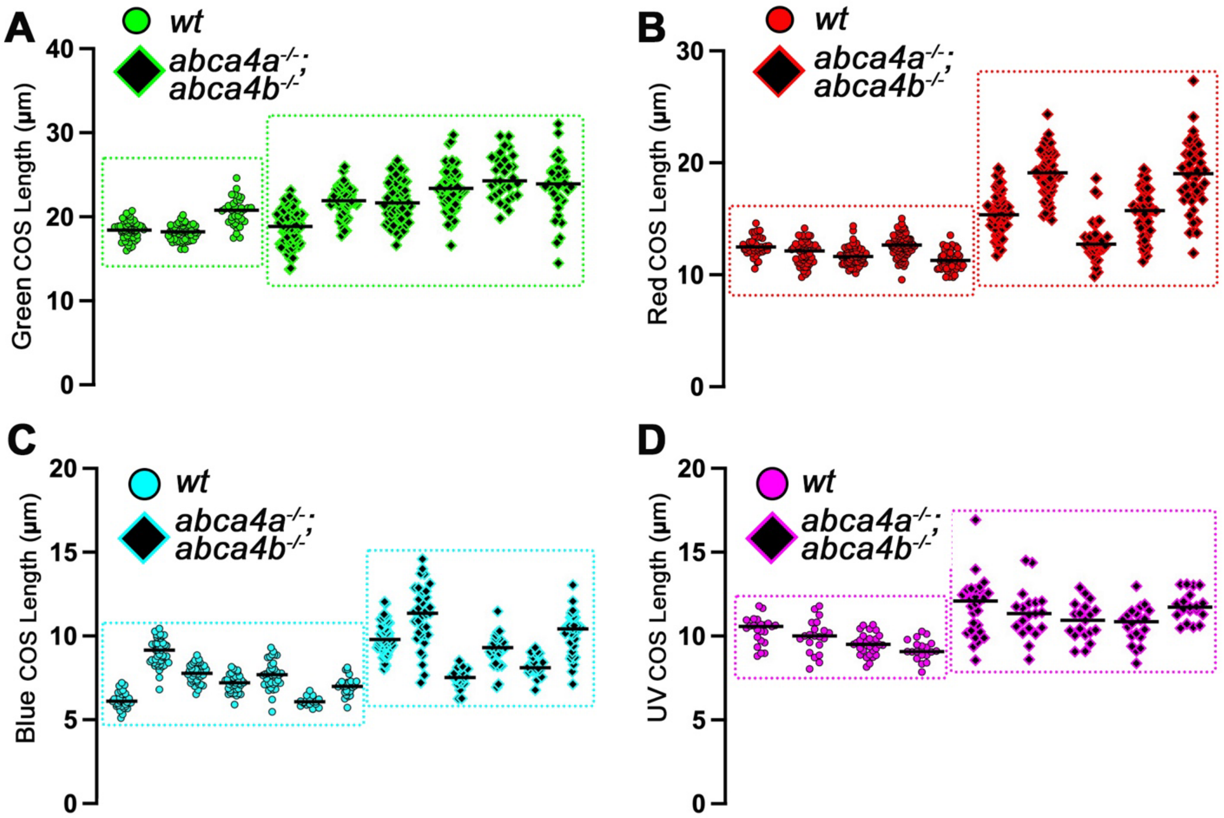

Fig. S4. Individual COS length measurements taken from 5-week *wildtype* and *abca4a*$^{ca30/ca30}$;*abca4b*$^{ca33/ca33}$ double mutants. (**A**) Green COS, (**B**) Red COS, (**C**) Blue COS, (**D**), UV COS.

Disease Models & Mechanisms • Supplementary information

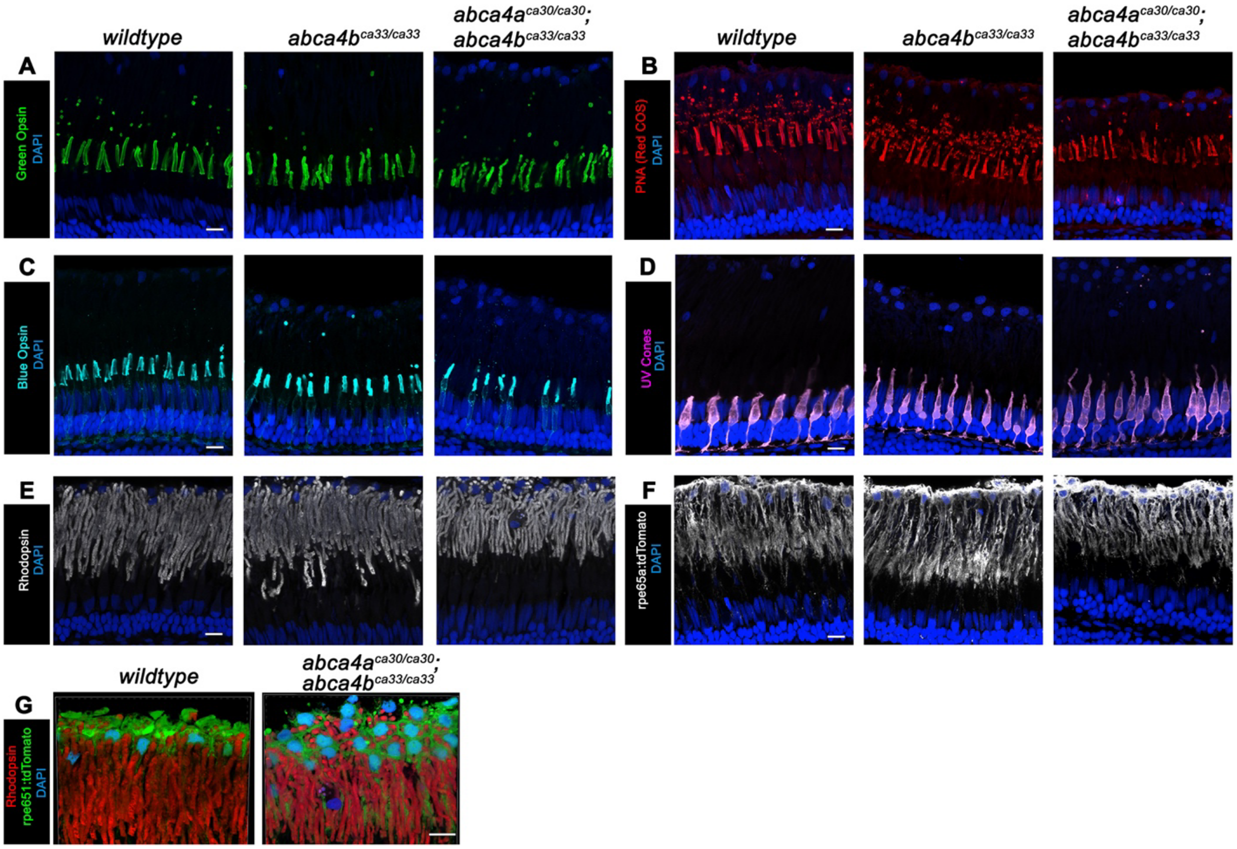

Fig. S5. Photoreceptors and RPE in 2 year *wild-type*, *abca4b*$^{-/-}$ and *abca4a*$^{-/-}$; *abca4b*$^{-/-}$ double mutant retina. Confocal z-projections of 2 year *wildtype*, *abca4b*$^{ca33/ca33}$ and *abca4*$^{ca30/ca30}$*;abca4b*$^{ca33/ca33}$ double mutant retina co-labeled with DAPI (blue) and (**A**) green COS labeled with anti-Green Opsin antibodies (green, z = 3.4 μm), (**B**) red COS with PNA (red, z = 3.6 μm), (**C**) blue COS with anti-Blue Opsin antibodies (blue, z = 4.4 μm), (**D**) UV cones expressing EGFP labeled with anti-GFP antibodies (violet, z = 4.4 μm), (**E**) ROS with anti-Rhodopsin antibodies (white, z = 1.74 μm), (**F**) RPE expressing tdTomato labeled with anti-RFP antibodies (white, z = 2.4 μm), (**G**) an oblique 3D-projection of ROS labeled with anti-Rhodopsin antibodies (red) with RPE expressing tdTomato (green) revealing ROS phagosomes in RPE cell bodies. Scale bars, 10 μm.

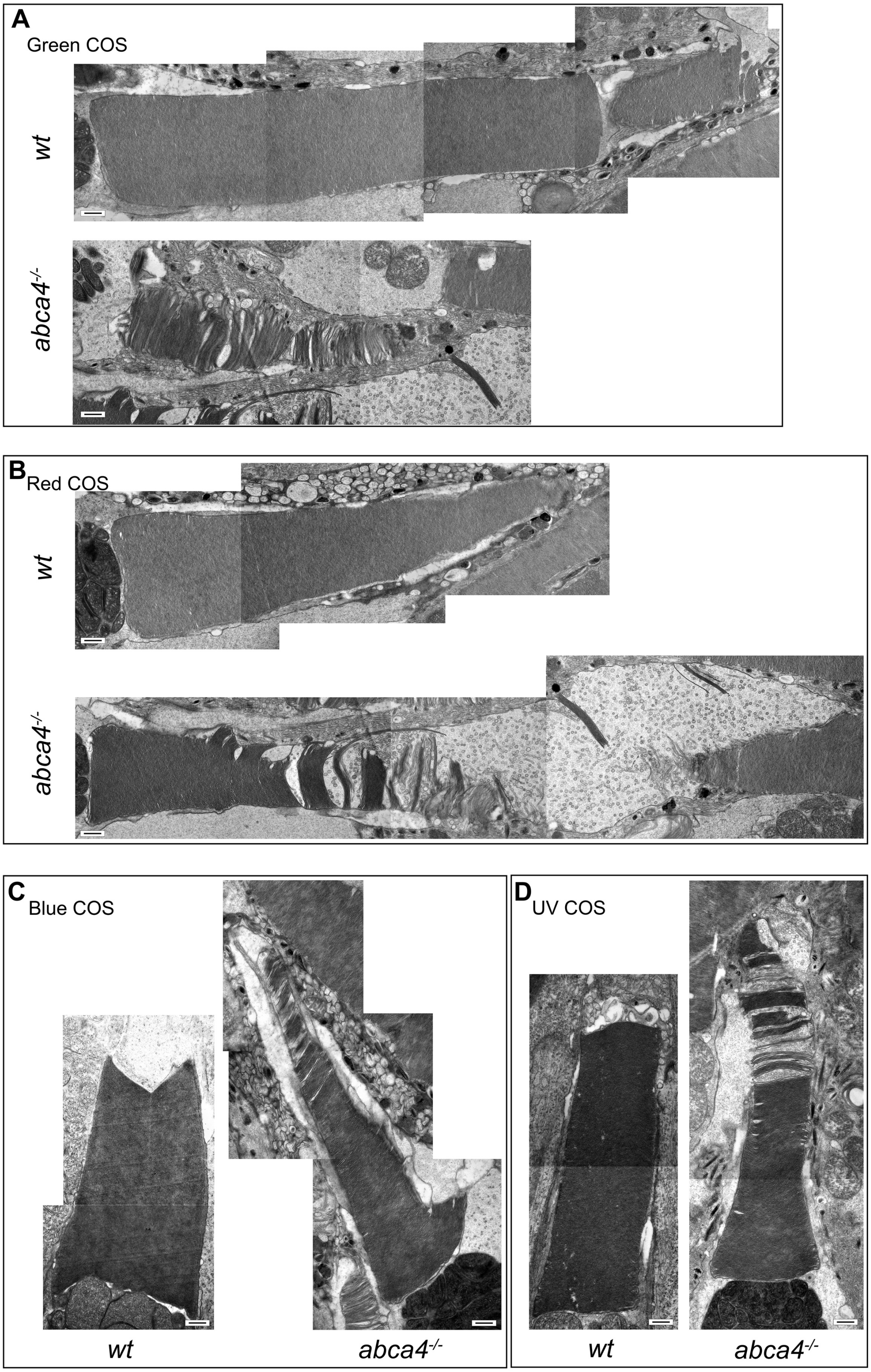

Fig S6. Ultrastructure of *abca4*$^{-/-}$ double mutant cone outer segments. TEM images (**A**) Green COS, (**B**) Red COS, (**C**) Blue COS, (**D**) UV COS in 4 month *wild-type* (*wt*) and *abca4*$^{-/-}$ (*abca4a*$^{ca31/ca31}$;*abca4b*$^{ca33/ca33}$) double mutant retina. Scale bars, 0.5 μm.

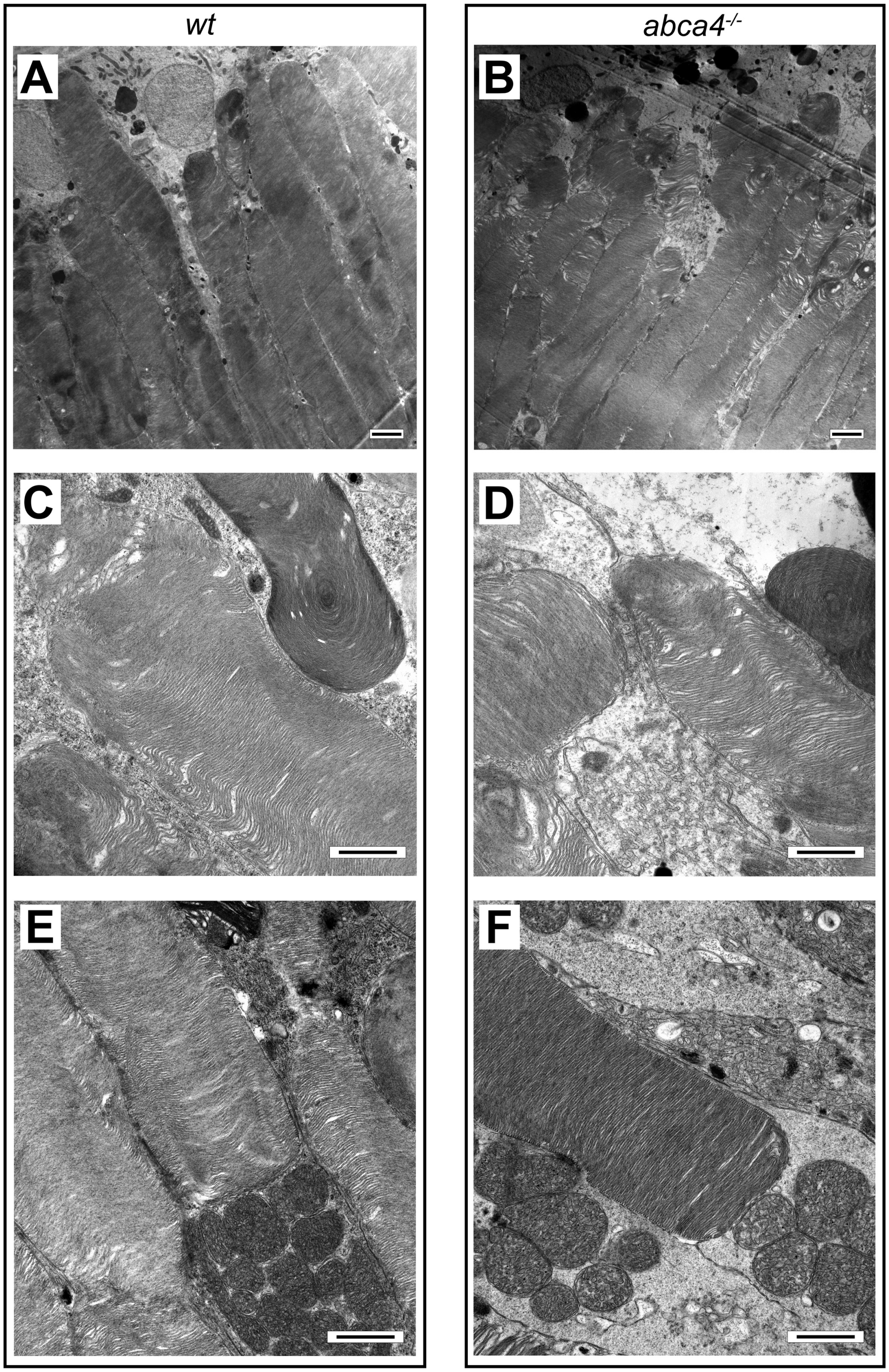

Fig S7. Ultrastructure of rod outer segments in *abca4* double mutant retina. TEM images of ROS in 4 month *wild-type* (*wt*) and *abca4*$^{-/-}$ (*abca4a*$^{ca31/ca31}$;*abca4b*$^{ca33/ca33}$) double mutant retina. Low magnification of *wt* (**A**) and (**B**) *abca4*$^{-/-}$ double mutant ROS (RPE at top of images). High magnification of the ROS tip in *wt* (**C**) and *abca4*$^{-/-}$ double mutant (**D**). Low magnification of the base of ROS in *wt* (**E**) and *abca4*$^{-/-}$ double mutant (**F**), the large mitochondria in the ellipsoid are seen below the ROS base. Scale bars, 2 μm (A, B), 1 μm (C-F).

Disease Models & Mechanisms • Supplementary information

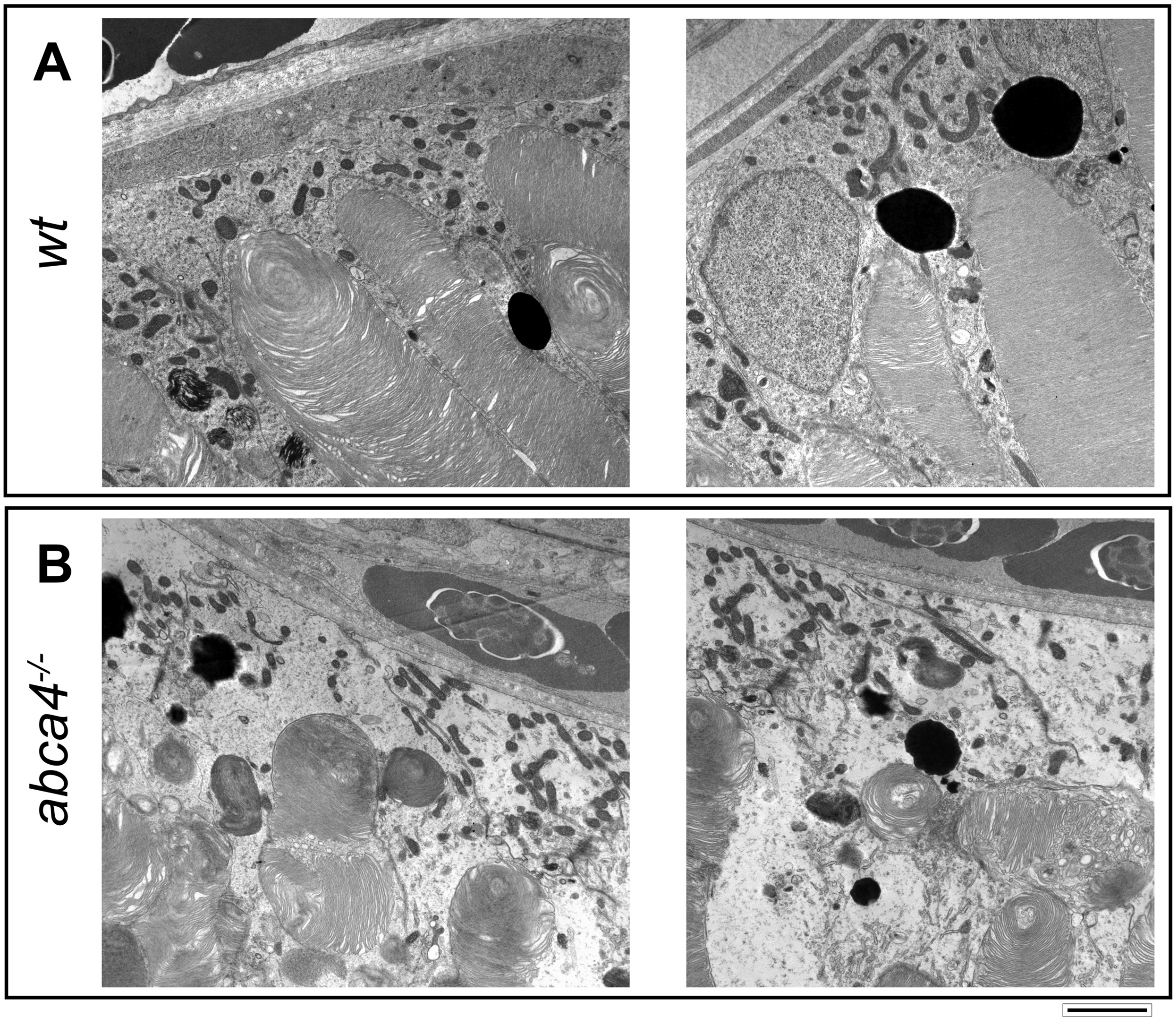

Fig S8. Ultrastructure of RPE in 4 month *abca4* double mutant retina. TEM images of (**A**) *wild-type* (*wt*) and (**B**) *abca4*$^{-/-}$ (*abca4a*$^{ca31/ca31}$;*abca4b*$^{ca33/ca33}$) double mutant RPE region. Scale bar, 2 μm.

Disease Models & Mechanisms • Supplementary information

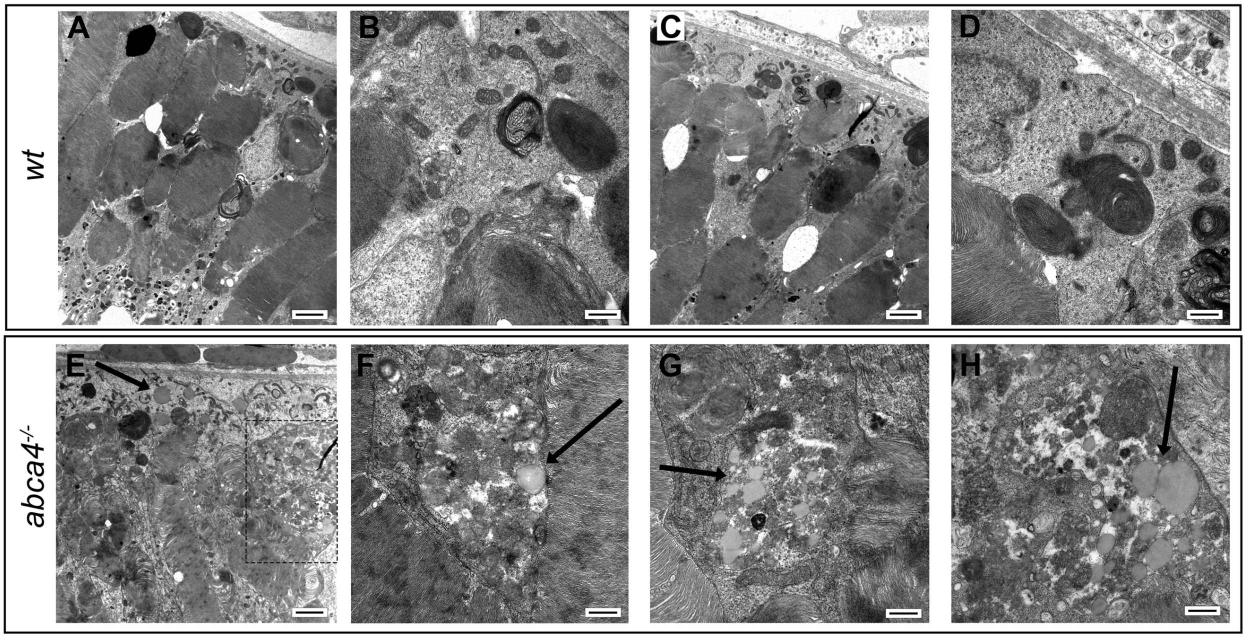

Fig. S9. Ultrastructure of RPE in 1 year *abca4* double mutant retina. TEM images of (**A-D**) *wild-type* (*wt*) and (**E-H**) *abca4*$^{-/-}$ (*abca4a*$^{ca31/ca31}$;*abca4b*$^{ca33/ca33}$) double mutant RPE region. The boxed area in (**E**) indicates a membrane bound structure that contains multiple lipofuscin droplets. Arrows (E-H) indicate lipofuscin. Scale bars, 2 μm (A, C, E), 0.5 μm (B, D, F-H).

Disease Models & Mechanisms • Supplementary information

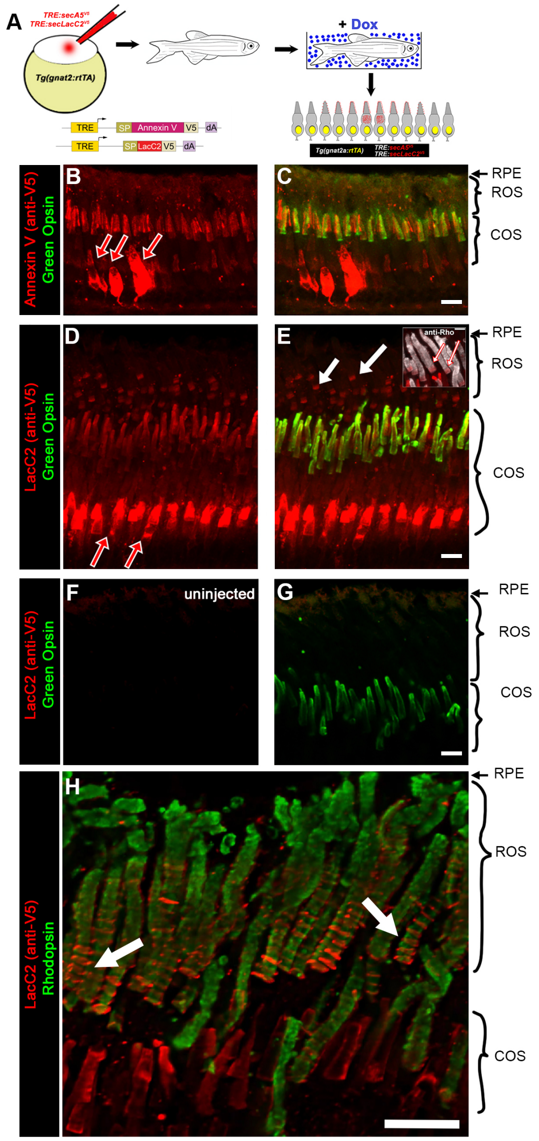

Fig. S10. In vivo labeling of externalized PS. (**A**) Injection of *TRE:secA5*^{V5} or *TRE:secLacC2*^{V5} pTol plasmid DNA and transposase mRNA into the pan-cone TetOn driver line, *Tg(gnat2:rtTA),* single cell embryo creates genetic mosaics in which small clones of cones express and secrete Annexin 5 (A5) or LacC2 following doxycycline (DOX)-induced gene expression. (**B**, **C**) Confocal z-projections (z = 6.4 μm) of retinal sections from 10 month *Tg(gnat2:rtTA)* injected with *TRE:secA5*^{V5} treated 2 days with DOX labeled with (**B**) anti-V5 antibodies and together with (**C**) anti-Green Opsin antibodies, 3-4 cones that express A5 are indicated by red arrows (B). Anti-V5 labels nearby COS (some green COS), and cone phagosomes distal to COS. (**D**, **E**) Retinal sections from 4 month *Tg(gnat2:rtTA)* injected with *TRE:secLacC2*^{V5} treated 3 days with DOX, labeled with anti-V5 antibodies (**D**) and together with anti-Green Opsin antibodies (**E**). The presumptive Golgi region of 3 cones that express LacC2 are indicated by red arrows. Anti-V5 labels nearby COS (some green COS), cone phagosomes distal to COS, and the base of rod outer segments. Inset (**E**) double-labeling of anti-Rhodopsin and anti-V5, arrows indicate V5 labeling at the base of rod outer segments. Confocal z-projections from 4 month uninjected *Tg(gnat2:rtTA)* treated 3 days with DOX labeled with anti-V5 antibodies (**F**) and together with anti-Green Opsin antibodies (**G**). (**H**) Deconvoluted confocal z-projection (z = 5.4 μm) from 5 month *Tg(gnat2:rtTA)* that had been injected with *TRE:secA5*^{V5} treated 14 days with DOX, labeled with anti-V5 and anti-Rhodopsin antibodies. White arrow indicate tilted ROS, where anti-V5 labeling appears as rings. Scale bars: A-G, 5 μm; H, 10 μm.

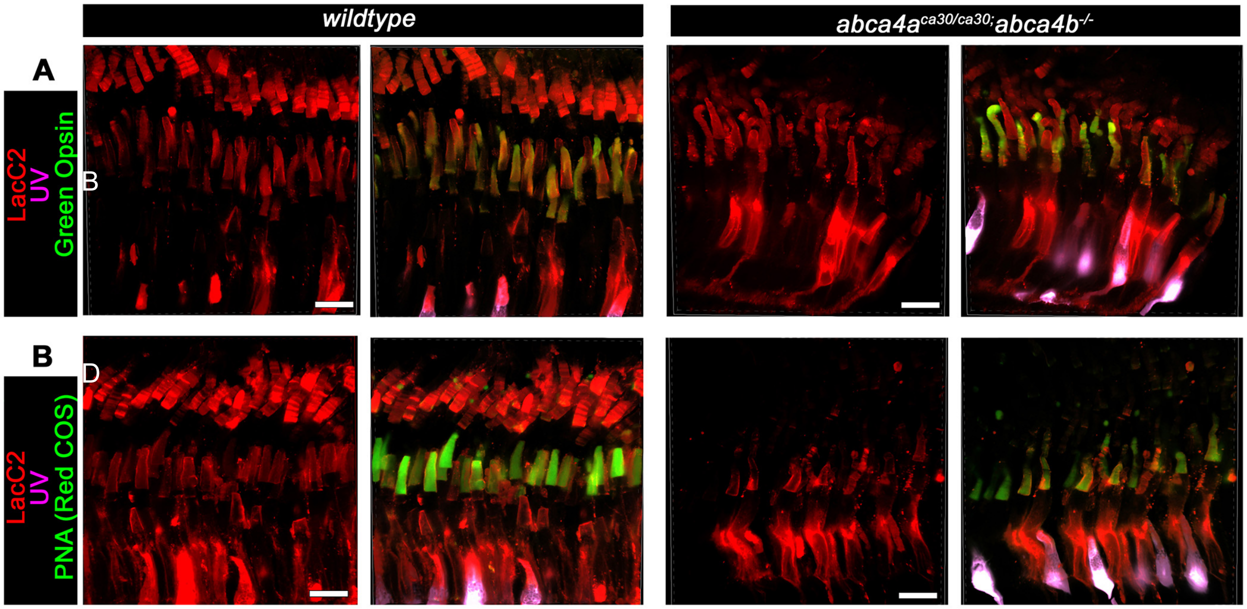

Fig. S11. In vivo labeling of externalized PS. Confocal z-projections (z = 4.4 μm) of the photoreceptor layer in *wildtype* and *abca4$^{ca30/ca30}$;abca4b$^{ca33/ca33}$* 4 month fish DOX-treated for 7 days. (**A**) Retina co-labeled with anti-V5 (LacC2, red) and anti-Green Opsin (green), and UV cones express EGFP (violet). (**B**) Retina co-labeled with anti-V5 (LacC2, red) and PNA (green), and UV cones express EGFP (violet). Scale bars, 10 μm.

Disease Models & Mechanisms • Supplementary information
